# Validation of a multiplexed immunoassay for immunological analysis of pre erythrocytic malaria vaccines

**DOI:** 10.1038/s41541-024-01039-z

**Published:** 2025-01-20

**Authors:** L. K. Stockdale, S. Provstgaard-Morys, D. Bellamy, D. Woods, K. Rapi, A. Bajer, B. Hollingdale, O. Muñoz, S. Malik, A. V. S. Hill, K. J. Ewer

**Affiliations:** 1 Jenner Institute, University of Oxford, and the NIHR Oxford Biomedical Research Centre, Oxford, OX3 7DQ UK; 2 Current affiliation: GSK Vaccines Institute for Global Health (Global Health Vaccines R&D), GSK, Siena, Italy

**Keywords:** Malaria, Antibodies, Peptide vaccines

## Abstract

The primary immunological readout for clinical trials of R21/MatrixM™ malaria vaccine, is total IgG antibody specific to the central four amino acid NANP repeat region of the circumsporozoite protein. A multiplexed assay, which includes NANP, was developed and validated for four antigens representing components of the R21 immunogen. Initial assay optimisation included validation of the HBsAg international standard. Further validation performed in Oxford covered intra and inter-assay, and inter-operator variability, accuracy of QC and standard curve material, and included bridging to a singleplex NANP6 ELISA. The assay was shown to be robust and specific, with a broad dynamic range. We report a strong linear relationship between NANP6 IgG as measured by the singleplex ELISA and the multiplexed assay with rho values of 0.89 and 0.88 for two separate clinical trials (both *p* < 0.0005). This assay can be used to measure antibodies specific to the CSP NANP repeat region, CSP C-term region, full length R21 and HBsAg.

## Introduction

Globally, malaria is one of the leading causes of mortality and morbidity. In 2022, an estimated 608,000 deaths were attributed to malaria, with the majority occurring in sub-Saharan Africa^1^. The burden of morbidity and mortality is primarily borne by pregnant women and young children, who account for approximately two-thirds of all malaria deaths.

R21 with adjuvant Matrix M™ (R21/MM) and RTS,S with adjuvant AS01 (RTS,S/AS01), are the only malaria vaccines recommended for use by the WHO^2,3^. Both vaccines target the pre-erythrocytic stage of *Plasmodium falciparum* (*Pf*) infection. At this stage of the *Pf* lifecycle, the parasite travels from the site of mosquito bite to the liver and utilises a surface protein—the circumsporozoite protein (CSP)—which covers the surface of the sporozoite, for hepatocyte invasion. The R21 vaccine construct comprises recombinant nanoparticles decorated with *Pf* CSP central repeat (Asparagine-Alanine-Asparagine-Proline (NANP)), and the C-terminal (C-term), fused to the Hepatitis B surface antigen (HBsAg). Both R21/MM and RTS,S/AS01 malaria vaccines use HBsAg as the core structural protein onto which CSP is attached. However, a key difference is that RTS,S has an excess of HBsAg compared with R21. Levels of IgG antibody specific to six NANP repeats (NANP6) is the primary immunological readout for R21/MM clinical trials. An association between increased anti-CSP IgG antibody titre and vaccine-induced protection against *P. falciparum* infection has been reported for both RTS,S/AS01^4^ and R21/MM^5^. A Phase 2b clinical trial of R21 in 450 Burkinabe children aged 5–17 months reported 12-month vaccine efficacy of 77% (95% CI 67–84) using a 0, 1, 2 month vaccine schedule^5^ and 78% (95% CI 71 to 83) after 2 years of follow up^6^. NANP6-specific IgG antibodies (measured by a singleplex ELISA) were significantly higher in children who did not have any clinical malaria episodes during both the first and second years of follow-up^6^. NANP6 is only one antigen contained within the R21 vaccine construct, and antibody responses to this antigen alone were measured in the Phase 2b clinical trial of R21/MM^5^. However, other antigens may play a role in the immune response, for example IgG avidity to the C-term has been implicated in protective efficacy of RTS,S^7^. Given this, and the need to use small volumes of paediatric samples sparingly, development of a multiplex assay where simultaneous measurement of antibody responses to multiple antigens was undertaken. With input from the commercial sponsor of R21/MM vaccine development, Meso Scale Discovery (MSD) was chosen to develop a bespoke four-plex assay where full length R21, C-term, HBsAg and NANP6 were included. This report details the validation of this assay prior to its use in a subsequent Phase 3 clinical trial of R21/MM conducted in five sites across four Sub-Saharan African countries (Burkina Faso, Mali, Tanzania and Kenya) in children aged 5–36 months. Twelve-month vaccine efficacy data from this study showed efficacy of 75% (95% CI 71-79 *p* < 0.0001) against multiple clinical malaria episodes at sites where vaccine administration was just before the malaria season, and efficacy of 68% (95% CI 61-74 *p* < 0.0001) at sites with year-round transmission^3^. The Phase 3 study reported fewer cases of malaria in the highest NANP6 IgG responders when NANP6 IgG levels were split into tertiles (tertile 1 = reference, tertile 3 = 0.52 Incidence Rate Ratio, IRR (95% CI 0.36–0.75), *p* = 0.001). Additionally, higher NANP6 IgG antibody titres and higher efficacy were reported in the younger (5–17 months) age group compared with older children (18–36-months). Time to first malaria episode efficacy was 78% (95% CI 73–82) in younger children and 70% (95% CI 64–74) in older children (*p* = 0024).

The development of robust and reliable immuno-assays is crucial for the evaluation of any vaccine. Assays must be sensitive, specific, and reproducible to ensure accurate assessment of the immune response elicited by the vaccine. Extensive work has been conducted to validate a CSP assay for RTS,S measuring IgG responses to the R32LR protein which comprises two NVDP oligopeptides and thirty NANP repeats linked to the dipeptide Leu-Arg (NVDP[NANP]152LR)^8^. However, there is still an ongoing requirement for the harmonisation and standardisation of immunogenicity assays. In their 2023 paper, Mugo et al. found a high degree of correlation between the singleplex NANP6 ELISA used in Oxford to measure antibody responses to R21/MM, the R32LR CSP ELISA used by GSK (alternatively named the “Ghent assay”) to measure responses to RTSS, and another singleplex (NANP)9 ELISA used in Kenya (between rho 0.91 and 0.97, all *p* < 0.0001)^9^.

Here, we describe the validation of a high-throughput multiplex MSD assay measuring NANP6, C-term, full-length R21, and HBsAg. This bespoke assay utilises 4-spot, 96-well plates coated with the 4 antigens detailed above, and an electrochemiluminescent (ECL) detection system to detect antigen-specific IgG in plasma or serum samples. Using SULFO-TAG™ conjugated anti-IgG detection antibodies, the system detects light emitted upon electrochemical stimulation when specific antibodies bind to antigen coated on the plate surface. In addition, we compare NANP6-specific IgG antibodies as measured by the multiplexed MSD assay, to the singleplex NANP6 ELISA used previously in Oxford R21/MM clinical trials. We demonstrate the robust performance of the multiplexed MSD assay and show good correlation between NANP6 IgG antibodies as measured by the singleplex and multiplexed assays.

## Results

### Initial method development

Meso Scale Discovery (MSD), Rockville, MD, USA conducted initial method development using 120 plasma and serum samples from 3 R21/MM clinical trials, comprising adults and children from Burkina Faso, and adults in the UK (Table 1). In summary, NANP6 and C-term peptides were BSA conjugated (for conjugation to BSA, peptides were modified with a terminal Cysteine) and demonstrated increased signal response with increased peptide concentration (see supplementary materials, Supplementary Note 2). Assessment of sample dilutions showed an optimal dilution ratio of 1:1000 for pre-vaccination timepoints (D0/D35) and 1:100,000 for post-vaccination timepoints. Standard curve material was comprised of a pool of highly responding RTS,S vaccinated UK CHMI participants at the timepoint of peak antibody response (4 weeks after 3rd dose of RTS,S). Initial dilution of standard curve material was 1:10,000 with 6 subsequent 4-fold dilutions. The eighth standard was diluent only. Three Quality Control (QC) samples were determined as: High (QC1: 1:40,000 dilution), Medium (QC2: 1:640,000 dilution) and Low (QC3: 1:10,240,000 dilution) and were made from the same material as the standard curve. Standard curve samples were run in duplicate and QC samples were run in quadruplicate. Sample matrix was investigated, with both plasma and serum requiring the same sample dilutions, and measurements being equally reproducible. Full details are available in supplementary materials (Supplementary Note 2). This method development demonstrated good coating uniformity across all antigens, high specificity with <1% non-specific binding, and good reproducibility of standards, controls, and clinical samples. Following this method development, further in-house validation was performed as detailed below.

**Table 1:** Summary of samples used in this validation

Study/Population	Clinical trials registration number	Group (vaccine dose)	Age	Sample timepoint	n	Sample type
VAC060 - Burkinabe adults	NCT02925403	group 1 - 10 μg R21/ 50 μg Matrix-M at d0, d28, d56	18-45 years	D0 (pre-vaccine), D84 (one month after final vaccine)	8	Serum
		group 3 - saline placebo	18-45 years	D0, D84 (both R21 unvaccinated)	4	Serum
VAC076 - Burkinabe infants	NCT03896724	group 1 - 10 μg R21/ 25 μg Matrix-M at d0, d28, d56	5-17 months	D0 (pre-vaccine), D84 (one month after final vaccine)	6	Plasma
		group 2 - 10 μg R21/ 50 μg Matrix-M at d0, d28, d56	5-17 months	D0 (pre-vaccine), D84 (one month after final vaccine)	5	Plasma
		group 3 - Rabies placebo at d0, d28, d56	5-17 months	D0, D84 (both R21 unvaccinated)	4	Plasma
VAC072 - UK adults	NCT03970993	grp 2 - 10 μg R21/50 μg Matrix-M d0, d28, d168	18-45 years	D196 (one month after final vaccine)	8	Serum and Plasma
		grp 3 - 10 μg R21/50 μg Matrix-M d0, d28, d56	18-45 years	D84 (one month after final vaccine)	21	Serum and Plasma
		grp 4 - 50 μg R21/50 μg Matrix-M d0, d28, and 10 μg R21/50 μg Matrix-M d168	18-45 years	D196 (one month after final vaccine)	7	Serum and Plasma
		grp 5 - 10 μg R21/50 μg Matrix-M d0, d28, and 2 μg R21/50 μg Matrix-M d168	18-45 years	D196 (one month after final vaccine)	10	Serum and Plasma
		grp 6 - unvaccinated controls	18-45 years	D0, D35 (both R21 unvaccinated)	5	Serum and Plasma

### In-house validation (Jenner Institute, Oxford)

**Fig. 1:**
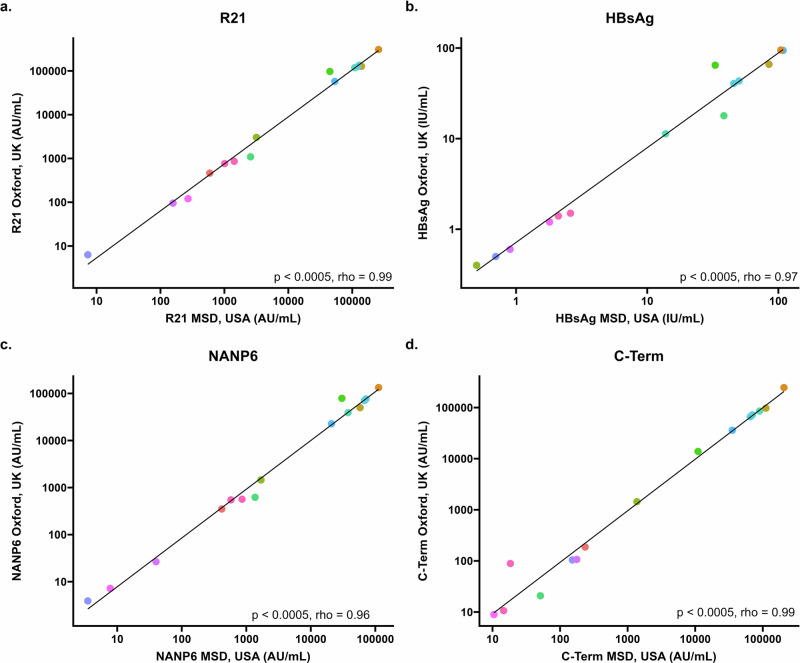
Correlations between clinical trials samples run on the MSD assay in Oxford, UK and MSD, USA. **a** Correlation between IgG response to full length R21 vaccine construct as measured by MSD in USA (x axis) and Oxford (y axis) using 15 samples from individuals before and following R21/MM vaccination. **b** Correlation between IgG response to hepatitis B surface antigen (HBsAg) as measured by MSD in USA (*x*-axis) and Oxford (*y*-axis) using 15 samples from individuals before and following R21/MM vaccination. **c** Correlation between IgG response to six repeats of the four amino acid sequence (NANP) as measured by MSD in USA (*x*-axis) and Oxford (*y*-axis) using 15 samples from individuals before and following R21/MM vaccination. **d** Correlation between IgG response to the C-terminus of CSP as measured by MSD in USA (x axis) and Oxford (y axis) using 15 samples from individuals before and following R21/MM vaccination. All figures show Spearman’s rank correlation coefficient.

To study inter-laboratory variability, standards (run in duplicate) and QC samples (run in quadruplicate) and *n* = 15 clinical trials samples (run in quadruplicate) were compared between one experiment carried out at MSD laboratories, Rockville, MD, USA and one experiment (plate 1) carried out at the Jenner Institute laboratories, Oxford, UK (Fig. 1). We found a mean coefficient of variation (CV) of 2.5% for all antigens for all 7 points of the standard curve, plus the blank (standard 8). Standard 7 (with a dilution of 1:40,960,000) was associated with the highest level of variation between laboratories (between 5.4% for HBsAg and 15.1% for C-term; Table 2a). For the QC samples, we found increasing variation with increased dilution for all antigens; a mean (across all antigens) of 14.1% CV for QC1, a mean of 17.3% CV for QC2 and a mean of 21.7% CV for QC3 (Table 2a). Clinical trial sample measurements were positively correlated between the two laboratories. This was statistically significant for all antigens (Fig. 1).

**Table 2:** a. Inter-laboratory comparison between Oxford, UK and MSD, USA of standard curve (STD1-8) and QC sample (High, Mid, Low) measurements, for each antigen. b. Inter-laboratory comparison between Oxford, UK and MSD, USA of unknown clinical samples (S1-15) of either plasma or serum, for each antigen

a												
Standard curve points									QC samples			
R21	Calculated Concentration (AU/mL)								R21	Calculated Concentration (AU/mL)		
	**STD 01**	**STD 02**	**STD 03**	**STD 04**	**STD 05**	**STD 06**	**STD 07**	**STD 08**		**High Control**	**Mid Control**	**Low Control**
**MSD, USA**	9.321	2.318	0.572	0.148	0.036	0.009	0.002	0.000	**MSD, USA**	1.145	0.300	0.075
**Oxford, UK**	9.631	2.316	0.573	0.145	0.036	0.009	0.003	0.000	**Oxford, UK**	0.886	0.214	0.049
**Mean**	9.476	2.317	0.572	0.146	0.036	0.009	0.002	0.000	**Mean**	1.016	0.257	0.062
**SD**	0.155	0.001	0.000	0.001	0.000	0.000	0.000	0.000	**SD**	0.130	0.040	0.010
**CV(%)**	1.6	0.0	0.1	0.9	1.2	2.2	11.9	0.0	**CV(%)**	12.8	16.8	21.3
**HBsAg**	**Calculated Concentration (IU/mL)**								**HBsAg**	**Calculated Concentration (IU/mL)**		
**MSD, USA**	0.02448	0.00598	0.00152	0.00037	0.00010	0.00002	0.00001	0.00000	**MSD, USA**	0.00320	0.00080	0.00020
**Oxford, UK**	0.02493	0.00604	0.00150	0.00038	0.00009	0.00002	0.00001	0.00000	**Oxford, UK**	0.00220	0.00050	0.00010
**Mean**	0.02471	0.00601	0.00151	0.00038	0.00010	0.00002	0.00001	0.00000	**Mean**	0.00270	0.00070	0.00020
**SD**	0.00022	0.00003	0.00001	0.00000	0.00000	0.00000	0.00000	0.00000	**SD**	0.00050	0.00010	0.00000
**CV(%)**	0.9	0.5	0.4	0.9	2.6	2.4	5.4	0.0	**CV(%)**	18.1	19.2	22.3
**NANP6**	**Calculated Concentration (AU/mL)**								**NANP6**	**Calculated Concentration (AU/mL)**		
**MSD, USA**	6.420	1.591	0.397	0.100	0.026	0.006	0.002	0.000	**MSD, USA**	0.761	0.199	0.050
**Oxford, UK**	6.596	1.593	0.397	0.100	0.024	0.006	0.002	0.000	**Oxford, UK**	0.616	0.146	0.033
**Mean**	6.508	1.592	0.397	0.100	0.025	0.006	0.002	0.000	**Mean**	0.689	0.173	0.041
**SD**	0.088	0.001	0.000	0.000	0.001	0.000	0.000	0.000	**SD**	0.070	0.030	0.010
**CV(%)**	1.3	0.1	0.0	0.1	3.1	0.0	10.3	0.0	**CV(%)**	10.5	15.4	20.6
**C-Term**	**Calculated Concentration (AU/mL)**								**C-Term**	**Calculated Concentration (AU/mL)**		
**MSD, USA**	3.247	0.786	0.197	0.050	0.013	0.003	0.001	0.000	**MSD, USA**	0.404	0.102	0.026
**Oxford, UK**	3.351	0.798	0.193	0.050	0.012	0.003	0.001	0.000	**Oxford, UK**	0.298	0.071	0.016
**Mean**	3.299	0.792	0.195	0.050	0.013	0.003	0.001	0.000	**Mean**	0.351	0.086	0.021
**SD**	0.052	0.006	0.002	0.000	0.000	0.000	0.000	0.000	**SD**	0.050	0.020	0.000
**CV(%)**	1.6	0.8	1.0	0.1	3.4	0.2	15.1	0.0	**CV(%)**	15.1	18	22.8

b															
R21	Serum							Plasma							
	Mean Calculated Concentration (AU/mL)														
	**S1 D0**	**S2 D0**	**S3 D35**	**S4 D35**	**S5 D84**	**S6 D84**	**S7 D196**	**S8 D196**	**S9 D0**	**S10 D35**	**S11 D0**	**S12 D35**	**S13 D84**	**S14 D196**	**S15 D196**
**MSD, USA**	590.2	3164.4	2564.3	1423.1	44667	53310.2	257973.2	128640.8	7.3	157.1	269.3	1009.7	109270.5	139348	120497.1
**Oxford, UK**	462	3027	1088.5	858.8	97013.2	57474.7	307364.4	134661.8	6.3	95.3	120.1	763	118307.1	127599.8	124086.6
**Mean**	526.1	3095.7	1826.4	1141	70840.1	55392.5	282668.8	131651.3	6.8	126.2	194.7	886.4	113788.8	133473.9	122291.8
**SD**	64.1	68.7	737.9	282.1	26173.1	2082.3	24695.6	3010.5	0.5	30.9	74.6	123.4	4518.3	5874.1	1794.7
**CV(%)**	12.2	2.2	40.4	24.7	36.9	3.8	8.7	2.3	7.7	24.5	38.3	13.9	4	4.4	1.5
**HBsAg**	**Serum**							**Plasma**							
	**Mean Calculated Concentration (IU/mL)**														
**MSD, USA**	0.5	0.5	38.4	2.6	33	108.7	104.4	50.1	0.7	0.9	1.8	2.1	13.8	84.8	45.7
**Oxford, UK**	0.4	0.4	17.9	1.5	64.6	94.1	95	43.3	0.5	0.6	1.2	1.4	11.3	66.1	40.6
**Mean**	0.4	0.4	28.1	2.1	48.8	101.4	99.7	46.7	0.6	0.8	1.5	1.8	12.5	75.4	43.2
**SD**	0.1	0	10.2	0.6	15.8	7.3	4.7	3.4	0.1	0.2	0.3	0.3	1.2	9.3	2.6
**CV(%)**	14.9	3	36.4	27.5	32.4	7.2	4.7	7.2	15.9	22.9	21.2	18.9	9.9	12.4	5.9
**NANP6**	**Serum**							**Plasma**							
	**Mean Calculated Concentration (AU/mL)**														
**MSD, USA**	417.6	1689.8	1367.6	860.1	30330	20920.8	112277.7	71707	3.5	39.9	7.8	578.6	37837.4	58080.8	67657.4
**Oxford, UK**	350.8	1451.6	620.9	565.3	78858.4	22663.2	133295	76523.3	3.9	26.6	7.2	547.8	39031.3	49828.8	70671.4
**Mean**	384.2	1570.7	994.3	712.7	54594.2	21792	122786.3	74115.1	3.7	33.2	7.5	563.2	38434.4	53954.8	69164.4
**SD**	33.4	119.1	373.3	147.4	24264.2	871.2	10508.7	2408.2	0.2	6.7	0.3	15.4	597	4126	1507
**CV(%)**	8.7	7.6	37.5	20.7	44.4	4	8.6	3.2	4.8	20	3.4	2.7	1.6	7.6	2.2
**C-Term**	**Serum**							**Plasma**							
	**Mean Calculated Concentration (AU/mL)**														
**MSD, USA**	234.8	1373.8	51.1	18.4	11013.6	35369.2	205952.9	70113.3	152.4	176.9	10.5	14.6	89665.5	111322	65486.3
**Oxford, UK**	186.9	1441.8	20.8	88.9	13851	35934.5	246179.3	71917.8	104.5	106.8	8.9	10.6	85422.4	97384.6	66152.1
**Mean**	210.9	1407.8	35.9	53.7	12432.3	35651.9	226066.1	71015.6	128.5	141.9	9.7	12.6	87543.9	104353.3	65819.2
**SD**	24	34	15.2	35.3	1418.7	282.7	20113.2	902.2	24	35	0.8	2	2121.5	6968.7	332.9
**CV(%)**	11.4	2.4	42.3	65.7	11.4	0.8	8.9	1.3	18.6	24.7	8.2	15.6	2.4	6.7	0.5

To study intra-run variability, standards, QC samples and clinical trials samples were compared between two plates (named plate 1 and plate 2 in Table 3a and b) run by the same operator on the same day in Oxford, UK. We found a mean CV of 7.8% for all antigens for all 8 points of the standard curve. Standard 7 was associated with the highest level of variability between plates (a mean of 38.5% CV; the lowest being 11.4% for HBsAg and the highest 53.6% for C-term) Table 3a. For the QC samples, we found increasing variability with increased dilution for all antigens; a mean of 0.9% CV for QC1, a mean of 3% CV for the QC2 and a mean of 6% CV for the QC3 (Table 3a). For clinical trials samples we see a mean variability of 2.5% CV for all samples over all antigens between two plates run on the same day by the same operator (Table 3b).

**Table 3:** a. Comparison of measurements of standard curve (STD1-8) and QC samples (High, Med, Low) conducted by the same operator on the same day (Intra-run), for each antigen. b. Comparison of measurements of unknown clinical samples (S1-15) of plasma and serum conducted by the same operator on the same day (Intra-run), for each antigen

a												
Standard curve points									QC samples			
R21	Calculated concentration (AU/mL)								R21	Calculated concentration (AU/mL)		
	**STD1**	**STD2**	**STD3**	**STD4**	**STD5**	**STD6**	**STD7**	**STD8**		**High**	**Med**	**Low**
**Plate 1**	9.565	2.183	0.597	0.146	0.037	0.009	0.002	0.000	**Plate 1**	0.862	0.211	0.049
**Plate 2**	10.689	2.281	0.576	0.141	0.032	0.008	0.006	0.000	**Plate 2**	0.849	0.194	0.042
**Mean**	10.127	2.232	0.587	0.143	0.035	0.009	0.004	0.000	**Mean**	0.855	0.202	0.046
**SD**	0.562	0.049	0.010	0.002	0.003	0.001	0.002	0.000	**SD**	0.006	0.008	0.003
**%CV**	5.6	2.2	1.8	1.7	8.3	6.5	45.3	0.0	**%CV**	0.8	4.1	7.3
**HBsAg**	**Calculated concentration (IU/mL)**								**HBsAg**	**Calculated concentration (IU/mL)**		
**Plate 1**	0.02490	0.00561	0.00157	0.00040	0.00010	0.00002	0.00001	0.00000	**Plate 1**	0.00222	0.00055	0.00013
**Plate 2**	0.02686	0.00575	0.00150	0.00038	0.00009	0.00002	0.00001	0.00000	**Plate 2**	0.00224	0.00053	0.00012
**Mean**	0.02588	0.00568	0.00153	0.00039	0.00009	0.00002	0.00001	0.00000	**Mean**	0.00223	0.00054	0.00012
**SD**	0.00098	0.00007	0.00004	0.00001	0.00000	0.00000	0.00000	0.00000	**SD**	0.00001	0.00001	0.00000
**%CV**	3.8	1.2	2.3	2.6	4.4	1.5	11.4	0.0	**%CV**	0.4	1.4	2.9
**NANP6**	**Calculated concentration (AU/mL)**								**NANP6**	**Calculated concentration (AU/mL)**		
**Plate 1**	6.491	1.517	0.410	0.101	0.025	0.007	0.001	0.000	**Plate 1**	0.605	0.143	0.033
**Plate 2**	7.272	1.581	0.401	0.095	0.022	0.006	0.004	0.000	**Plate 2**	0.580	0.131	0.027
**Mean**	6.882	1.549	0.406	0.098	0.024	0.006	0.003	0.000	**Mean**	0.592	0.137	0.030
**SD**	0.390	0.032	0.005	0.003	0.002	0.000	0.001	0.000	**SD**	0.012	0.006	0.003
**%CV**	5.7	2.1	1.1	3.2	7.7	7.3	43.7	0.0	**%CV**	2.1	4.7	8.8
**C-Term**	**Calculated concentration (AU/mL)**								**C-Term**	**Calculated concentration (AU/mL)**		
**Plate 1**	3.329	0.755	0.199	0.051	0.013	0.003	0.001	0.000	**Plate 1**	0.294	0.070	0.016
**Plate 2**	3.735	0.789	0.188	0.048	0.012	0.003	0.002	0.000	**Plate 2**	0.291	0.067	0.014
**Mean**	3.532	0.772	0.194	0.049	0.012	0.003	0.002	0.000	**Mean**	0.293	0.068	0.015
**SD**	0.203	0.017	0.005	0.002	0.001	0.000	0.001	0.000	**SD**	0.001	0.001	0.001
**%CV**	5.7	2.2	2.8	3.2	4.7	8.5	53.6	0.0	**%CV**	0.4	2.1	5.2

b															
R21	Serum							Plasma							
	Mean Calculated Concentration (AU/mL)														
	**S1**	**S2**	**S3**	**S4**	**S5**	**S6**	**S7**	**S8**	**S9**	**S10**	**S11**	**S12**	**S13**	**S14**	**S15**
	**D0**	**D0**	**D35**	**D35**	**D84**	**D84**	**D196**	**D196**	**D0**	**D35**	**D0**	**D35**	**D84**	**D196**	**D196**
**Plate 1**	206.1	1451.0	508.5	365.8	48633.6	30731.3	302463.9	131806.2	4.8	124.7	74.6	740.3	123095.4	112404.7	126771.6
**Plate 2**	222.9	1527.1	546.1	354.0	46967.5	31504.2	318318.2	128751.6	4.7	126.5	74.5	731.1	128256.2	116971.2	127511.4
**Mean**	214.5	1489.0	527.3	359.9	47800.6	31117.8	310391.1	130278.9	4.8	125.6	74.5	735.7	125675.8	114688.0	127141.5
**SD**	8.4	38.1	18.8	5.9	833.1	386.4	7927.1	1527.3	0.1	0.9	0.0	4.6	2580.4	2283.3	369.9
**%CV**	3.9	2.6	3.6	1.6	1.7	1.2	2.6	1.2	1.1	0.7	0.0	0.6	2.1	2.0	0.3
	**Serum**							**Plasma**							
**HBsAg**	**Mean Calculated Concentration (IU/mL)**														
**Plate 1**	0.15993	0.17504	8.58431	0.62257	35.06768	53.27558	92.78758	40.30427	0.38235	1.13463	0.47278	1.21553	62.85877	10.06528	40.98924
**Plate 2**	0.16973	0.18060	9.33036	0.65711	34.57818	58.02617	94.88451	40.93406	0.40146	1.13484	0.50124	1.29180	65.21666	10.32135	39.60525
**Mean**	0.16483	0.17782	8.95734	0.63984	34.82293	55.65088	93.83604	40.61917	0.39191	1.13473	0.48701	1.25366	64.03771	10.19332	40.29724
**SD**	0.00490	0.00278	0.37303	0.01727	0.24475	2.37530	1.04846	0.31490	0.00956	0.00011	0.01423	0.03813	1.17894	0.12804	0.69200
**%CV**	3.0	1.6	4.2	2.7	0.7	4.3	1.1	0.8	2.4	0.0	2.9	3.0	1.8	1.3	1.7
	**Serum**							**Plasma**							
**NANP6**	**Mean Calculated Concentration (AU/mL)**														
**Plate 1**	154.8	577.8	288.5	247.4	37740.6	11511.8	130233.5	69852.5	3.4	6.1	19.8	513.6	47804.1	35253.2	71662.2
**Plate 2**	166.5	645.7	301.1	251.4	37087.2	11736.3	132480.4	69718.1	3.1	7.2	19.1	533.6	47430.5	38030.3	72664.7
**Mean**	160.6	611.8	294.8	249.4	37413.9	11624.0	131357.0	69785.3	3.2	6.7	19.5	523.6	47617.3	36641.7	72163.4
**SD**	5.8	34.0	6.3	2.0	326.7	112.3	1123.5	67.2	0.1	0.5	0.4	10.0	186.8	1388.5	501.2
**%CV**	3.6	5.5	2.1	0.8	0.9	1.0	0.9	0.1	4.4	8.2	1.8	1.9	0.4	3.8	0.7
	**Serum**							**Plasma**							
**C-Term**	**Mean Calculated Concentration (AU/mL)**														
**Plate 1**	89.4	754.7	9.7	4.6	7056.7	19273.6	242842.4	67730.6	82.9	7.1	85.7	7.9	98409.0	82144.9	69889.6
**Plate 2**	92.2	803.2	9.1	5.0	7031.8	21822.2	269559.8	71983.1	89.8	8.3	88.7	9.1	104997.7	88240.7	70839.5
**Mean**	90.8	778.9	9.4	4.8	7044.2	20547.9	256201.1	69856.9	86.3	7.7	87.2	8.5	101703.3	85192.8	70364.5
**SD**	1.4	24.3	0.3	0.2	12.5	1274.3	13358.7	2126.3	3.5	0.6	1.5	0.6	3294.4	3047.9	475.0
**%CV**	1.5	3.1	2.7	4.7	0.2	6.2	5.2	3.0	4.0	7.9	1.7	7.1	3.2	3.6	0.7

**Fig. 2:**
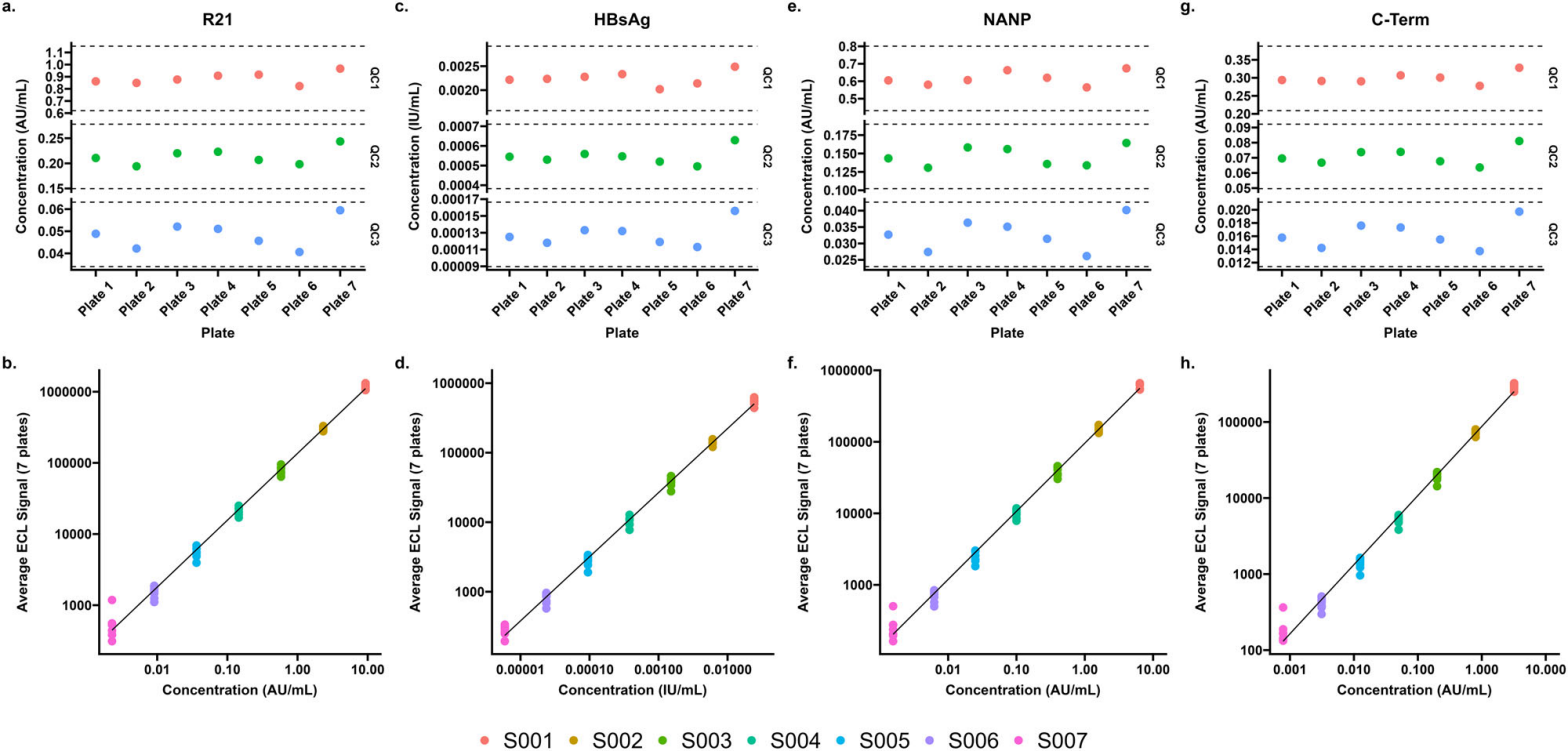
Concentrations for intra-run, Inter-run, and Inter-operator for each antigen included in the multiplex MSD assay. Data for 7 MSD plates used to calculate variability. **a**, **c**, **e**, and **g** - Control (QC) samples for each of 7 plates with ± 30% thresholds for each sample. **b**, **d**, **f**, and **h** - Standards 1-7 of the standard curve for each of the 7 plates in AU/ml for full length R21 antigen, IgG in IU/mL for Hepatitis B surface antigen, IgG levels in AU/mL for NANP6 antigen, IgG levels in AU/mL for C-terminus antigen.

To study inter-run variability, the mean of standards, QC samples and clinical trials samples for two plates run by the same operator on the same day over three days in Oxford, UK were compared (mean of plates 1 and 2 compared to the mean of plates 3 and 4 compared to the mean of plates 5 and 6; Fig. 2). We found a mean CV of 2.6% across all antigens and all 7 points of the standard curve. Standard 7 was associated with the highest level of variability between plates (a mean of 5.3% CV; the lowest being 2.7% for R21 and the highest 7.6% for HBsAg) Table 4a. As before, for the QC samples, we found increasing variability with increased dilution for all antigens; a mean of 2.6% CV for QC1, a mean of 3.9% CV for the QC2 and a mean of 5.5% CV for the QC3 (Table 4a).

**Table 4:** a. Comparison of measurements of standard curve (STD1-8) and QC samples (High, Med, Low) conducted by the same operator on different days (Inter-run), for each antigen. b. Comparison of measurements of unknown clinical samples (S1-15) of plasma and serum conducted by the same operator on different days (Inter-run), for each antigen

a												
Standard curve points									QC samples			
R21	Calculated concentration (AU/mL)								R21	Calculated concentration (AU/mL)		
	**STD1**	**STD2**	**STD3**	**STD4**	**STD5**	**STD6**	**STD7**	**STD8**		**High**	**Med**	**Low**
**Day 1**	9.565	2.183	0.597	0.146	0.037	0.009	0.002	0.000	**Day 1**	0.862	0.211	0.049
**Day 2**	9.890	2.266	0.557	0.145	0.036	0.009	0.002	0.000	**Day 2**	0.877	0.220	0.052
**Day 3**	9.305	2.386	0.576	0.142	0.036	0.009	0.002	0.000	**Day 3**	0.917	0.207	0.046
**Mean**	9.586	2.279	0.576	0.144	0.036	0.009	0.002	0.000	**Mean**	0.885	0.213	0.049
**SD**	0.239	0.083	0.017	0.002	0.001	0.000	0.000	0.000	**SD**	0.023	0.006	0.003
**%CV**	2.5	3.7	2.9	1.1	2.0	0.2	2.7	0.0	**%CV**	2.6	2.6	5.4
**HBsAg**	**Calculated concentration (IU/mL)**								**HBsAg**	**Calculated concentration (IU/mL)**		
**Day 1**	0.02490	0.00561	0.00157	0.00040	0.00010	0.00002	0.00001	0.00000	**Day 1**	0.00222	0.00055	0.00013
**Day 2**	0.02603	0.00599	0.00149	0.00035	0.00010	0.00002	0.00001	0.00000	**Day 2**	0.00228	0.00056	0.00013
**Day 3**	0.02417	0.00623	0.00151	0.00037	0.00010	0.00002	0.00001	0.00000	**Day 3**	0.00202	0.00052	0.00012
**Mean**	0.02503	0.00594	0.00152	0.00037	0.00010	0.00002	0.00001	0.00000	**Mean**	0.00217	0.00054	0.00013
**SD**	0.00076	0.00026	0.00003	0.00002	0.00000	0.00000	0.00000	0.00000	**SD**	0.00011	0.00002	0.00001
**%CV**	3.0	4.3	2.2	5.4	0.3	2.5	7.6	0.0	**%CV**	5.1	3.0	4.6
**NANP6**	**Calculated concentration (AU/mL)**								**NANP6**	**Calculated concentration (AU/mL)**		
**Day 1**	6.491	1.517	0.410	0.101	0.025	0.007	0.001	0.000	**Day 1**	0.605	0.143	0.033
**Day 2**	6.873	1.560	0.379	0.102	0.024	0.007	0.002	0.000	**Day 2**	0.606	0.158	0.036
**Day 3**	6.375	1.631	0.408	0.097	0.025	0.006	0.002	0.000	**Day 3**	0.620	0.136	0.031
**Mean**	6.580	1.569	0.399	0.100	0.025	0.006	0.002	0.000	**Mean**	0.610	0.146	0.033
**SD**	0.213	0.047	0.014	0.002	0.001	0.000	0.000	0.000	**SD**	0.007	0.009	0.002
**%CV**	3.2	3.0	3.5	2.0	3.2	4.2	6.4	0.0	**%CV**	1.1	6.4	6.2
**C-Term**	**Calculated concentration (AU/mL)**								**C-Term**	**Calculated concentration (AU/mL)**		
**Day 1**	3.329	0.755	0.199	0.051	0.013	0.003	0.001	0.000	**Day 1**	0.294	0.070	0.016
**Day 2**	3.375	0.776	0.189	0.051	0.013	0.003	0.001	0.000	**Day 2**	0.290	0.074	0.018
**Day 3**	3.183	0.822	0.198	0.049	0.013	0.003	0.001	0.000	**Day 3**	0.301	0.068	0.016
**Mean**	3.296	0.784	0.195	0.050	0.013	0.003	0.001	0.000	**Mean**	0.295	0.070	0.016
**SD**	0.082	0.028	0.004	0.001	0.000	0.000	0.000	0.000	**SD**	0.004	0.003	0.001
**%CV**	2.5	3.6	2.1	1.7	0.7	3.2	4.7	0.0	**%CV**	1.5	3.6	5.7

b															
	Serum							Plasma							
R21	Mean Calculated Concentration (AU/mL)														
	**S1**	**S2**	**S3**	**S4**	**S5**	**S6**	**S7**	**S8**	**S9**	**S10**	**S11**	**S12**	**S13**	**S14**	**S15**
	**D0**	**D0**	**D35**	**D35**	**D84**	**D84**	**D196**	**D196**	**D0**	**D35**	**D0**	**D35**	**D84**	**D196**	**D196**
**Day 1**	206.1	1451.0	508.5	365.8	48633.6	30731.3	302463.9	131806.2	4.8	124.7	74.6	740.3	123095.4	112404.7	126771.6
**Day 2**	251.8	1491.8	558.9	348.1	52725.9	30696.4	293736.2	135844.1	7.1	131.7	104.1	826.9	133017.7	121402.8	127070.8
**Day 3**	508.4	3505.6	1194.8	707.2	108113.7	61281.8	361956.5	149189.3	6.0	107.8	94.7	742.5	126368.8	111669.7	115795.8
**Mean**	228.9	1471.4	533.7	356.9	50679.8	30713.8	298100.1	133825.2	6.0	128.2	89.4	783.6	128056.5	116903.8	126921.2
**SD**	22.8	20.4	25.2	8.8	2046.1	17.5	4363.8	2019.0	1.2	3.5	14.8	43.3	4961.2	4499.1	149.6
**%CV**	10.0	1.4	4.7	2.5	4.0	0.1	1.5	0.0	1.0	3.0	2.0	4.0	6.0	7.0	8.0
	**Serum**							**Plasma**							
**HBsAg**	**Mean Calculated Concentration (IU/mL)**														
**Day 1**	0.15993	0.17504	8.58431	0.62257	35.06768	53.27558	92.78758	40.30427	0.38235	1.13463	0.47278	1.21553	62.85877	10.06528	40.98924
**Day 2**	0.21883	0.39802	9.46975	0.72629	38.63467	54.24248	93.65705	45.72908	0.51302	1.20676	0.64252	1.55893	73.42298	11.68473	43.56269
**Day 3**	0.37620	0.39338	18.85216	1.42632	74.56928	107.13730	105.56490	45.25933	0.49927	1.19765	0.58915	1.41685	61.77255	10.24013	36.00785
**Mean**	0.18938	0.28653	9.02703	0.67443	36.85118	53.75903	93.22231	43.01668	0.44769	1.17069	0.55765	1.38723	68.14087	10.87501	42.27597
**SD**	0.02945	0.11149	0.44272	0.05186	1.78350	0.48345	0.43474	2.71240	0.06534	0.03607	0.08487	0.17170	5.28210	0.80973	1.28673
**%CV**	15.5	38.9	4.9	7.7	4.8	0.9	0.5	0.0	1.0	3.0	2.0	4.0	6.0	7.0	8.0
	**Serum**							**Plasma**							
**NANP6**	**Mean Calculated Concentration (AU/mL)**														
**Day 1**	154.8	577.8	288.5	247.4	37740.6	11511.8	130233.5	69852.5	3.4	6.1	19.8	513.6	47804.1	35253.2	71662.2
**Day 2**	202.0	736.8	329.2	242.5	44047.3	12162.1	129158.2	77060.2	4.4	7.2	30.3	601.3	51616.6	39401.7	73304.0
**Day 3**	383.2	1848.5	651.6	498.1	83511.9	23208.5	151596.5	84610.5	4.0	6.8	27.4	544.3	46429.6	35434.9	62440.7
**Mean**	178.4	657.3	308.9	244.9	40893.9	11836.9	129695.8	73456.4	3.9	6.7	25.0	557.4	49710.3	37327.4	72483.1
**SD**	23.6	79.5	20.4	2.4	3153.3	325.1	537.7	3603.9	0.5	0.5	5.2	43.8	1906.3	2074.2	820.9
**%CV**	13.2	12.1	6.6	1.0	7.7	2.7	0.4	0.0	1.0	3.0	2.0	4.0	6.0	7.0	8.0
	**Serum**							**Plasma**							
**C-Term**	**Mean Calculated Concentration (AU/mL)**														
**Day 1**	89.4	754.7	9.7	4.6	7056.7	19273.6	242842.4	67730.6	82.9	7.1	85.7	7.9	98409.0	82144.9	69889.6
**Day 2**	103.1	712.8	12.2	6.6	8008.6	19623.9	218479.4	70246.8	110.8	10.1	117.1	11.7	95592.9	81583.4	64509.2
**Day 3**	195.2	1550.1	23.2	13.0	15030.4	38427.8	283702.1	78323.7	105.7	9.7	114.0	12.0	92352.2	80990.4	59960.7
**Mean**	96.3	733.7	10.9	5.6	7532.6	19448.8	230660.9	68988.7	96.9	8.6	101.4	9.8	97001.0	81864.1	67199.4
**SD**	6.8	20.9	1.3	1.0	476.0	175.1	12181.5	1258.1	14.0	1.5	15.7	1.9	1408.0	280.7	2690.2
**%CV**	7.1	2.8	11.8	17.9	6.3	0.9	5.3	0.0	1.0	3.0	2.0	4.0	6.0	7.0	8.0

For clinical trials samples we see a mean variability of 5.3% CV for any sample for any antigen between three plates run on different days (Table 4b).

To study inter-operator variability, standards, QC samples and clinical trials samples were compared between plates run by different operators (first operator plate named plate 6 and second operator plate named plate 7 in Fig. 2a, c, e, g) on the same day in Oxford, UK. We found a mean CV of 1.8% for all antigens for all 7 points of the standard curve. Standard 7 was associated with the highest level of variability between plates (mean of 4.5% CV; the lowest being 3.9% for C-term and the highest 5.0% for R21, Table 5a). As before, for the QC samples, we found increasing variability with increased dilution for all antigens; a mean of 8.2% CV for QC1, a mean of 11.1% CV for the QC2 and a mean of 18.5% CV for the QC3 (Table 5a).

**Table 5:** a. Comparison of measurements of standard curve (STD1-8) and QC samples (High, Med, Low) conducted by the different operators on the same day (Inter-operator), for each antigen. b. Comparison of measurements of unknown clinical samples (S1-15) of plasma and serum conducted by different operators on the same day (Inter-operator), for each antigen

a												
Standard curve points									QC samples			
R21	Calculated concentration (AU/mL)								R21	Calculated concentration (AU/mL)		
	**STD1**	**STD2**	**STD3**	**STD4**	**STD5**	**STD6**	**STD7**	**STD8**		**High**	**Med**	**Low**
**Operator 1**	9.410	2.368	0.574	0.143	0.036	0.009	0.002	0.000	**Operator 1**	0.823	0.198	0.041
**Operator 2**	9.260	2.392	0.555	0.151	0.035	0.010	0.002	0.000	**Operator 2**	0.966	0.244	0.059
**Mean**	9.335	2.380	0.564	0.147	0.035	0.009	0.002	0.000	**Mean**	0.894	0.221	0.050
**SD**	0.075	0.012	0.010	0.004	0.000	0.000	0.000	0.000	**SD**	0.072	0.023	0.009
**%CV**	0.8	0.5	1.7	2.5	0.4	4.1	5.0	0.0	**%CV**	8.0	10.2	18.8
**HBsAg**	**Calculated concentration (IU/mL)**								**HBsAg**	**Calculated concentration (IU/mL)**		
**Operator 1**	0.02422	0.00614	0.00153	0.00038	0.00010	0.00002	0.00001	0.00000	**Operator 1**	0.00214	0.00050	0.00011
**Operator 2**	0.02410	0.00639	0.00144	0.00039	0.00009	0.00002	0.00001	0.00000	**Operator 2**	0.00249	0.00063	0.00016
**Mean**	0.02416	0.00626	0.00148	0.00038	0.00009	0.00002	0.00001	0.00000	**Mean**	0.00231	0.00056	0.00013
**SD**	0.00006	0.00013	0.00005	0.00001	0.00000	0.00000	0.00000	0.00000	**SD**	0.00017	0.00007	0.00002
**%CV**	0.3	2.0	3.3	2.2	2.3	5.3	4.8	0.0	**%CV**	7.5	11.8	16.0
**NANP6**	**Calculated concentration (AU/mL)**								**NANP6**	**Calculated concentration (AU/mL)**		
**Operator 1**	6.366	1.636	0.396	0.102	0.024	0.006	0.002	0.000	**Operator 1**	0.565	0.134	0.026
**Operator 2**	6.406	1.613	0.385	0.104	0.025	0.006	0.002	0.000	**Operator 2**	0.674	0.164	0.040
**Mean**	6.386	1.625	0.391	0.103	0.024	0.006	0.002	0.000	**Mean**	0.619	0.149	0.033
**SD**	0.020	0.011	0.005	0.001	0.000	0.000	0.000	0.000	**SD**	0.055	0.015	0.007
**%CV**	0.3	0.7	1.4	1.3	1.0	2.1	4.5	0.0	**%CV**	8.8	10.2	21.1
**C-Term**	**Calculated concentration (AU/mL)**								**C-Term**	**Calculated concentration (AU/mL)**		
**Operator 1**	3.345	0.809	0.194	0.048	0.012	0.003	0.001	0.000	**Operator 1**	0.278	0.064	0.014
**Operator 2**	3.291	0.835	0.188	0.050	0.012	0.003	0.001	0.000	**Operator 2**	0.328	0.081	0.020
**Mean**	3.318	0.822	0.191	0.049	0.012	0.003	0.001	0.000	**Mean**	0.303	0.072	0.017
**SD**	0.027	0.013	0.003	0.001	0.000	0.000	0.000	0.000	**SD**	0.025	0.009	0.003
**%CV**	0.8	1.5	1.5	1.5	0.3	0.1	3.9	0.0	**%CV**	8.3	12.0	17.9

b															
R21	Serum							Plasma							
	**S1**	**S2**	**S3**	**S4**	**S5**	**S6**	**S7**	**S8**	**S9**	**S10**	**S11**	**S12**	**S13**	**S14**	**S15**
	**D0**	**D0**	**D35**	**D35**	**D84**	**D84**	**D196**	**D196**	**D0**	**D35**	**D0**	**D35**	**D84**	**D196**	**D196**
**Operator 1**	467.0	3321.6	1192.7	734.9	97394.8	56548.9	332098.3	134555.9	5.5	116.8	101.8	829.3	131879.4	120785.6	123976.7
**Operator 2**	3577.2	2871.7	1033.8	1572.1	91825.8	56403.4	247352.4	129576.9	8.1	105.6	109.7	679.4	117859.7	120928.7	124990.9
**Mean**	2022.1	3096.7	1113.2	1153.5	94610.3	56476.2	289725.3	132066.4	6.8	111.2	105.7	754.4	124869.6	120857.2	124483.8
**SD**	1555.1	224.9	79.4	418.6	2784.5	72.7	42372.9	2489.5	1.3	5.6	3.9	74.9	7009.8	71.5	507.1
**%CV**	76.9	7.3	7.1	36.3	2.9	0.1	14.6	1.9	19.8	5.1	3.7	9.9	5.6	0.1	0.4
**HBsAg**	**Serum**							**Plasma**							
**Operator 1**	0.38135	0.49525	19.44798	1.41729	67.32264	96.66270	101.33569	42.54522	0.45690	1.14892	0.57235	1.48420	65.96699	10.25280	40.28771
**Operator 2**	1.58308	0.39167	16.98906	2.23866	68.10278	102.37685	81.77958	44.63772	0.53992	1.12014	0.63433	1.40301	66.43109	13.06976	44.72797
**Mean**	0.98221	0.44346	18.21852	1.82798	67.71271	99.51978	91.55764	43.59147	0.49841	1.13453	0.60334	1.44361	66.19904	11.66128	42.50784
**SD**	0.60087	0.05179	1.22946	0.41069	0.39007	2.85708	9.77806	1.04625	0.04151	0.01439	0.03099	0.04060	0.23205	1.40848	2.22013
**%CV**	61.2	11.7	6.7	22.5	0.6	2.9	10.7	2.4	8.3	1.3	5.1	2.8	0.4	12.1	5.2
**NANP6**	**Serum**							**Plasma**							
**Operator 1**	345.8	1507.1	690.4	467.5	78294.5	21487.0	140500.1	74685.5	2.6	5.2	26.4	542.8	49426.6	37340.6	68331.6
**Operator 2**	1806.1	1419.4	596.5	971.5	75078.6	23632.1	107745.2	77760.9	5.3	10.4	30.8	490.0	49386.2	43184.3	71608.1
**Mean**	1076.0	1463.3	643.4	719.5	76686.5	22559.6	124122.6	76223.2	4.0	7.8	28.6	516.4	49406.4	40262.4	69969.8
**SD**	730.1	43.8	47.0	252.0	1607.9	1072.6	16377.4	1537.7	1.3	2.6	2.2	26.4	20.2	2921.9	1638.3
**%CV**	67.9	3.0	7.3	35.0	2.1	4.8	13.2	2.0	33.3	33.3	7.6	5.1	0.0	7.3	2.3
**C-Term**	**Serum**							**Plasma**							
**Operator 1**	196.3	1588.1	19.6	9.9	13502.3	35632.3	274584.3	72447.3	104.6	7.2	109.6	9.6	101421.4	87747.8	64102.2
**Operator 2**	2649.3	1414.5	23.0	402.0	13258.6	35415.1	196920.3	70450.5	119.9	10.5	115.8	12.0	91340.5	88834.4	69089.7
**Mean**	1422.8	1501.3	21.3	205.9	13380.5	35523.7	235752.3	71448.9	112.2	8.9	112.7	10.8	96380.9	88291.1	66595.9
**SD**	1226.5	86.8	1.7	196.0	121.9	108.6	38832.0	998.4	7.6	1.7	3.1	1.2	5040.4	543.3	2493.7
**%CV**	86.2	5.8	8.0	95.2	0.9	0.3	16.5	1.4	6.8	18.7	2.7	10.9	5.2	0.6	3.7

For clinical trials samples we see a mean variability of 13.9% CV for any sample for any antigen between two different operators on the same day. The highest variability was seen in low titre pre-vaccination samples (D0 and D35 (Table 5b)).

### Investigating negative thresholds for multiplexed MSD

Fifty-eight pre-vaccination samples from UK volunteers enrolled into Controlled Human Malaria Infection (CHMI) studies (VAC055, 059, 065 and 072) were used to investigate negative thresholds. Enrolment exclusion criteria for these studies included a history of clinical malaria and travel to a malaria endemic region within 6 months of enrolment^10^.

The thresholds were calculated as the mean AU/ml from 58 pre-vaccination samples from UK adults, plus three standard deviations. For NANP this was 448.14 AU/ml, for C-term was 159.27 AU/ml, and for full length R21 it was 546.37 AU/ml. We did not use these thresholds as cut-offs in the data presented here.

### Bridging from standardised NANP6 ELISA to MSD

**Fig. 3:**
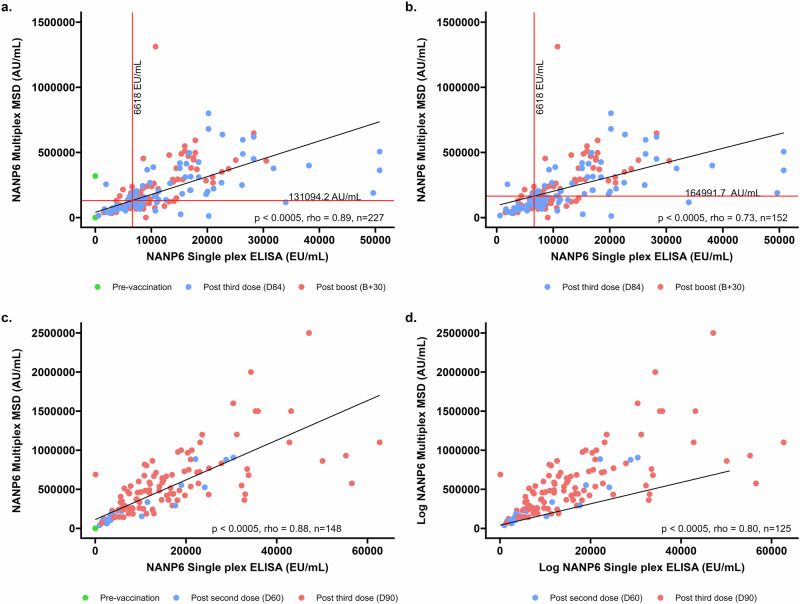
Correlations between total IgG to NANP6 as measured by multiplexed MSD and singleplex ELISA. **a** D0 (pre-vaccination samples, *n* = 75), D84 (1 month post primary series, *n* = 82) and B + 30 (1 month post booster dose given at 1 year, *n* = 70) for VAC076, **b** D84 (1 month post primary series, *n* = 82) and B + 30 (1-month post booster dose given at 1 year, *n* = 70) for VAC076. **c** SCRN (pre-vaccination samples, *n* = 23), D60 (1-month post 2 doses of R21/MM, *n* = 23), and D90 (1 month post primary series of 3 doses of R21/MM, *n* = 102) for VAC088, **d** D60 (1-month post 2 doses of R21/MM, *n* = 23), and D90 (1 month post primary series of 3 doses of R21/MM, *n* = 102) for VAC088. The value of 6618EU/ml is shown as a vertical line and the corresponding level measured by multiplex MSD is shown as a horizonal line for VAC076 samples only.

Bridging between a singleplex in-house NANP6 ELISA to the multiplex MSD assay was conducted using two cohorts; a Burkinabe infant cohort from a Phase 2b study (VAC076), and a Malian infant cohort from a Phase 1 clinical trial (VAC088). We conducted assay bridging to determine assay linearity, with and without pre-vaccination samples, and to interpolate a putative protective value on the multiplex MSD assay derived from the protective antibody titre of 6618EU/ml determined using the in-house singleplex NANP6 ELISA, as reported in Burkinabe infants in the Phase 2b clinical trial (VAC076)^5^ (Fig. 3a, b).

The in-house NANP6 singleplex ELISA^9^ uses a negative threshold, whereby any sample from a participant living in a malaria-endemic area, run at 1:500 dilution with an OD reading <0.2 OD is assigned an arbitrary value of 1 ELISA unit (EU).

There is a high degree of correlation between the two assays, with rho values of 0.89 and 0.88 (both *p* < 0.0005) for both studies used to investigate this when pre-vaccination samples are included (Fig. 3a; VAC076 and Fig. 3c; VAC088). When pre-vaccination samples are excluded, rho values are 0.73 and 0.80 (both *p* < 0.0005) respectively for VAC076 (Fig. 3b) and VAC088 (Fig. 3d).

To directly compare the singleplex and multiplex assays, we performed an interpolation using the NANP6 IgG titre of 6618 EU/mL found to be associated with a 77% vaccine efficacy in the R21 Phase 2b study^5^. Among samples from vaccinated individuals (Fig. 3b; VAC076) the interpolated value of NANP6 IgG as measured by the multiplexed MSD assay is 164991.7 AU/mL.

Using a linear regression model in which both studies are forced through the origin, the slopes of each study were compared. A one unit increase in MSD AU/ml resulted in a 0.045 increase in ELISA EU/ml for VAC076 and a 0.027 increase in ELISA EU/ml for VAC088. The two slopes differed from each other (*p* < 0.001).

In summary, we showed successful bridging of the in-house singleplex NANP6 ELISA to the multiplex MSD assay across two clinical trials, demonstrating a strong linear relationship between the assays. Additionally, we showed a difference in correlation between the two assays in two distinct clinical trials in different geographical locations.

To investigate the dynamic ranges of each assay, we took the ratio of the highest and lowest standards in the calibration curve, using raw ECL or OD signal for MSD and ELISA respectively. We report a dynamic range of 2250 for NANP6 IgG as measured by multiplex MSD (using an average from 12 MSD plates, the highest standard gives a signal of 606275.8 and the lowest standard, 269.5). The dynamic range for NANP6 IgG singleplex ELISA is 37 (using an average from 661 ELISA plates, the highest standard is 3.7 and the lowest standard is 0.1 OD).

## Discussion

This validation report covers the key performance characteristics also evaluated in several reported ELISA methods for novel vaccines^8,12,13^, and demonstrates that this multiplexed MSD assay fulfils the criteria for precision, linearity, specificity, robustness, non-interference and stability as per guidelines produced by the ICH and other regulatory authorities^14,15^.

This multiplexed assay allows for simultaneous measurement of antibody levels to multiple R21 vaccine antigens. Given the small sample volumes required, and the ability to combine antigens, this assay is particularly suited to clinical trials where limited biological material is available (such as paediatric populations), giving an advantage over many singleplex assays. Using pre-vaccination samples from malaria-naive volunteers, we have investigated negative thresholds for full length R21, C-term, and NANP6 antigens which can be used as potential cut-offs in future clinical trial analyses. Currently no negative threshold is used in analysis of MSD data. In this manuscript we investigate the use of samples from adult malaria naïve vaccine clinical trials volunteers for calculation of a possible negative threshold. The small number (*n* = 58) of malaria-naïve samples available to calculate a negative threshold highlights a potential limitation. Given that volunteers from malaria-endemic countries may have been previously exposed to malaria, pre-vaccination antibody data from non-UK participants is not investigated as a potential negative threshold here.

Assay bridging between the NANP6 singleplex ELISA (used to measure vaccine-specific antibody levels in the R21 Phase 2b clinical trial in Burkina Faso^9^), and NANP as measured by the multiplexed MSD assay (that is used for the Phase 3 clinical trial of R21 in Burkina Faso, Tanzania, Mali and Kenya^3^) showed strong linearity within individual clinical trials. However, we report differences between singleplex and multiplex assay relationship between the clinical trials. The clinical trials used to study the relationship between the single- and multiplex assays were conducted in different populations and ages. One study used to investigate assay bridging was conducted in Burkinabe children aged between 5 and 17 months at first vaccination whereas the other used samples from children aged 5–36 months and from Mali. These different assay correlations may be an artefact of the different clinical trial populations and ages used here, or might be indicative of context-dependent differences in relationships between the two assays described here. While this will not affect clinical trials conducted for R21/MM which report immunogenicity using either singleplex or multiplex NANP6 ELISA data, this finding does highlight that care should be taken when comparing data measured using different assays.

Some limitations of the multiplexed assay include the high sample dilutions required, the high cost, and the data management capacity required to handle the large quantities of data generated by this assay. While the assay has flexibility to add more antigens (up to a maximum of 10 per well), this cannot be done by the end user and would require re-validation, with associated costs, by the assay manufacturer. Although not discussed in this report, quality-checking, handling, cleaning and analysis of data resulting from multiplexed assays is not trivial and requires a step-change for laboratories used to dealing with singleplex assays. This assay shows acceptable inter-laboratory, intra- and inter-run and inter-operator variability in our hands in a small-scale validation scenario. Given the limitations described above, the MSD assay may not be appropriate for large scale analysis in field laboratories

In conclusion, this multiplex MSD assay is useful in large scale clinical trials when thousands of samples are analysed. The MSD platform allows concurrent measurement of four R21 vaccine antigens; full length R21, C-term, NANP6 and HBsAg which forms the core of the R21 nanoparticle. We have shown this assay to be sensitive and robust in our hands when data are analysed over different laboratories, operators, and days. Both serum and plasma can be used, and very small sample volumes are required, however the assay is expensive and must be manufactured by MSD which may limit its use in large field trials outside of licensure scenario. The use of this validated multiplexed assay will support further immunological studies of the R21 vaccine.

## Methods

### Ethics statement

Informed consent was obtained from all subjects in the original studies. The patients/participants provided their written informed consent to participate in these studies. Boards that approved study protocols include Oxford Vaccine Centre Biobank protocols, OXTREC, and local ethics in Burkina Faso and Mali for non-UK studies. UK study protocols were approved by the UKMHRA.

### Antigens

R21/MM vaccine comprises 18 repeats of NANP amino acid sequence. For both historic reasons, and to maintain comparability to previous clinical trials conducted by the Jenner Institute, six repeats of the four amino acid NANP sequence with a terminal cysteine are used in serological assays to measure CSP repeat region-specific antibody levels. This sequence has been previously described by Stoute et al. as demonstrating superior inhibition of Mab2A10 compared to both a monovalent CSP peptide and synthetic mimitope^16^. Additionally, measurements of NANP specific IgG responses using peptide of six, 12 and 19 NANP repeats have been shown to have no difference when tested on 200 clinical trial samples from 40 Burkinabe infants (VAC076, Supplementary Fig. 27).

Four antigens were selected for the multiplex MSD assay described here; a synthetic peptide corresponding to the *Pf* CSP C-term antigen produced by ProImmune, Oxford, UK; six repeats of the four amino acid NANP repeat with a cysteine residue added to the C-terminus (NANP6C) produced by ProImmune, Oxford, UK; full length R21 vaccine antigen; and recombinant HBsAg (both produced by Serum Institute of India Pvt. Ltd). Sequences for C-term, NANP6, and R21 can be found in the supplementary materials (Supplementary Note 3). With the exception of HBsAg, all antigen sequences were derived from the 3D7 reference strain of *Plasmodium falciparum*.

NANP6C and *Pf* CSP C-term peptides produced by ProImmune were generated from standard Fmoc chemistry via solid phase peptide synthesis. The peptides were synthesised from Fmoc protected amino acids on pre-loaded Wang resin. R21 is a recombinant fusion protein comprised of a portion of the *Pf* CSP fused with HBsAg and expressed as nanoparticles in recombinant yeast produced in *Hansenula polymorpha*. HBsAg was produced as described in Valenzula et al.^17^.

### Clinical samples used

120 serum and plasma samples from three R21 vaccine clinical trials (VAC060 (NCT02925403), VAC072 (NCT03970993) and VAC076 (NCT03896724)) were sent to MSD, Rockville, MD, USA for initial assay method development. These 120 samples were taken from both R21/MM vaccinated, and placebo vaccinated individuals. Details of samples can be found in Table 1.

Standard curve and QC material consisted of pooled sera from 33 high NANP6 responder participants (as measured by NANP6 singleplex ELISA) from a UK controlled human challenge study (CHMI; VAC055 NCT01883609, one month after a three-dose schedule of RTS,S).

Inter-laboratory correlations between results from the multiplex MSD assay carried out at MSD, Rockville, MD, USA and Jenner Institute laboratories, Oxford, UK were conducted on 15 samples from VAC060 (NCT02925403) and VAC072 (NCT03970993) (Table S1).

Negative thresholds were calculated using *n* = 58 pre-vaccination samples from Controlled Human Malaria Infection (CHMI) studies conducted in UK adults; VAC055 (NCT01883609), VAC059 (NCT02252640), VAC065 (NCT02905019), and VAC072 (NCT03970993).

Correlations between NANP6 as measured by the MSD assay and an in-house singleplex NANP6 ELISA were conducted on sample taken at pre-vaccination, one month following three doses of R21/MM and 1 month following booster dose (given at 1 year after primary three dose series) from a clinical trial conducted in Burkinabe children (VAC076; NCT03896724) and Malian children (VAC088; NCT05155579).

### Initial method development

According to EMA guidelines, the minimal metrics that need to be tested in order to validate an assay include identifying the characteristics of the analyte; identifying and selecting an appropriate platform; performing experiments to evaluate assay performance characteristics; and documenting the assay development procedure including any control strategy^18^. To that end, initial assay optimisation was conducted at Meso Scale Discovery LLC, Maryland, USA using 120 clinical trials samples (Table 1). This included antigen conjugation to BSA for peptide antigens NANP6 and C-term, optimisation of antigen coating concentration, blocking conditions, accuracy, precision, analytical sensitivity, specificity, dilution linearity and stability. This assay validation follows ICH guidelines^14^ and demonstrates precision, linearity, specificity, robustness, non-interference and stability. A tabulated overview of the ICH validation requirements can be found in Table S23.

In addition, the following parameters were investigated and optimised: testing of reference standard material, plate lot variability, sample dilution linearity, determination of dilutions for clinical trial samples, development of a standard curve from reference material, protocol optimisation, and assay reproducibility.

The Second International Standard for anti-hepatitis B surface antigen (anti-HBs) immunoglobulin, human (07/164) was used to assign a value of 243 IU/mL to the standard curve material. The concentration of HBsAg in the human reference standard serum sample was measured by back-fitting signals from the human reference standard serum sample to the HBsAg standard curve. Standard curve values used for concentration assignment were selected based on signals falling within the linear part of the anti-HB standard curve (Supplementary Table 12).

### Subsequent method development

Following initial assay optimisation, the MSD assay was tested in Oxford to ensure acceptable levels of variability were seen in our laboratories. Using standard curve (in duplicate) and QC samples and 15 clinical trials samples (in quadruplicate) (Supplementary Note 1; Supplementary Table 1), variability between laboratory sites, between plates, between days, and between operators was investigated.

To test inter-laboratory variability, we compared standards, QC samples and 15 clinical trials samples in one experiment run at MSD, Rockville, MD, USA, to one experiment run (by a different operator) in Oxford, UK.

To measure intra-run variability, we compared data from two plates run by the same operator on the same day in Oxford, UK.

Inter-run variability was investigated by comparing data from three different days, run by the same operator in Oxford, UK.

Inter-operator variability was tested by comparing data from two plates, run by two different operators on the same day in Oxford, UK.

Serum and plasma samples were diluted according to their timepoint and stored diluted in 2 batches to prevent repeated freeze/thaw cycles. Each batch contained sufficient sample to run 5 plates. Sample dilution batch 1 was used for 2 plates on Day 1 (plates 1 and 2), and 2 plates on Day 2 (plates 3 and 4). Sample dilution batch 2 was used for 2 plates on Day 3 (plates 5 and 6). The final plate (plate 7) which was run by a second operator, had separate dilutions made by that operator on the day of testing (Table 6).

**Table 6:** Summary of Oxford validation plan to test intra-run, inter-run and inter-operator variability

Dilution batch number	Plate Numbers	Day of Testing	Operator
1	1, 2	Day 1	Operator 1
1	3, 4	Day 2	Operator 1
2	5, 6	Day 3	Operator 1
Separate dilutions	7	Day 3	Operator 2

### Assay bridging

Data from NANP6 as measured by the multiplex MSD assay were compared to data from the Oxford laboratory singleplex NANP6 ELISA. Data from two R21/MM vaccine clinical trials (VAC076; NCT03896724 Burkinabe infants and VAC088; NCT05155579 Malian infants) were compared. Details of the singleplex NANP6 ELISA can be found in Mugo et al.^9^.

### NANP peptide length comparisons

To compare differences in NANP specific IgG measurements between six, 12 and 19 repeat lengths, ELISA was performed following the ‘Oxford-R21’ method described in Mugo et al.^9^ using NANP peptides of six, 12, and 19 NANP repeats.

### Initial method development

#### Investigating negative threshold

A putative negative threshold was investigated for use in the MSD assay using pre-vaccination (Day 0) samples from UK malaria-naive Controlled Human Malaria Infection (CHMI) participants (*n* = 58). A threshold of negative response was determined by taking the mean plus three standard deviations of the mean for each of R21, C-term and NANP6. HBsAg positivity is determined by anti-HBsAg levels being >10 mIU/mL as defined by the WHO^11^.

### Statistics

For measures of variation, the percent coefficient of variability (%CV) was calculated. %CV between assays run in different laboratories (inter-laboratory) on the same day (intra-run variability), on different days (inter-run variability), and between different operators (inter-operator variability) were calculated. A CV value of <20% was considered acceptable.

### Correlation analyses

Spearman’s rank correlation coefficient was used to correlate data from the same samples measured in two different laboratories (Fig. 1) in addition to samples measured using the singleplex and multiplex assays (Fig. 3a–d).

### Comparisons between correlations

To investigate potential differences in assay measurements between singleplex and multiplex measurements of NANP6 across clinical trials, a linear regression model without an intercept was employed, and only included samples taken post R21 vaccination. The model included ELISA titre as the independent variable, MSD titre as the dependent variable, and trial (VAC076 and VAC088), as an interaction term. Using the model output, a linear hypothesis test was performed to test for differences in the relationship between the assay measurements between the two studies.

All statistical analyses including interpolation between assays, were performed using Excel, R v4.2.2, RStudio 2023.06.0, and STATA v14.0 (Austin, Texas).

## Supplementary information

Supplementary information

## Data Availability

All data referred to in this manuscript are available either in tables and figures in the main text or in the Supplementary Materials
