# Supplementary material for: Validation of a multiplexed immunoassay for immunological analysis of pre erythrocytic malaria vaccines
Source: NPJ Vaccines. 2025 Jan 20;10:14. doi: 10.1038/s41541-024-01039-z (PMC11747258; doi:10.1038/s41541-024-01039-z)
Supplement: Supplementary file 1 — Supplementary information [file 41541_2024_1039_MOESM1_ESM.pdf]

Validation of a multiplexed immunoassay for immunological analysis of pre erythrocytic malaria vaccines

Stockdale L K^*[1], Provstgaard-Morys S*[1], Bellamy D[1], Woods D[1], Rapi K[1], Bajer A[1], Hollingdale B[1], Muñoz O[1], Malik S[1], Hill A S[1], Ewer K J[1,2]

*These authors contributed equally.

^ Corresponding author (lisa.stockdale@ndm.ox.ac.uk)

[1] Jenner Institute, University of Oxford, and the NIHR Oxford Biomedical Research Centre, Oxford OX3 7DQ, UK.

[2] Current affiliation: GSK Vaccines Institute for Global Health (Global Health Vaccines R&D), GSK, Siena, Italy

Supplementary Material

Supplementary Note 1 2
Clinical trial samples used in assay validation in Oxford 2
Supplementary Note 2 3
Comparison of BSA-conjugated and native peptides 3
Determine an optimal dilution of human reference serum sample for plate uniformity testing 4
Assess coating uniformity and signal at each antigen concentration using a single reference sample dilution 6
Assess coating uniformity and signal of each antigen at a single reference sample dilution 8
Assess signals and calculated concentrations of serum and plasma samples at two dilutions for each sample and Comparison of results from the production plates to the titration plates 9
Assessing the specificity of each assay 15
Concentration Assignment of HBsAg 16
Evaluate protocol incubation times and blocking conditions 17
Evaluate signal, concentration and recovery at 5 dilutions ranging from 100-fold to 1,000,000-fold in 10-fold steps for all 120 serum and plasma samples and to select the recommended sample dilution factors 25

Reproducibility: Evaluate accuracy (% recovery) and precision (%CVs) of each assay 32
Supplementary Note 3 41
Final Assay Protocol 41
Peptide Sequences 41
ICH Q14 Guideline Requirements for Analytical Procedure Validation 42

Supplementary Note 1

Clinical trial samples used in assay validation in Oxford

Supplementary Table 1 – Samples run in assay validation in Oxford

Sample	Timepoint	Sample type	Clinical trial	Clinical trial and vaccination dose details
S1	D0	Serum	VAC060	Burkina Faso adult
S2	D0	Serum	VAC060	Burkina Faso adult
S3	C+35	Serum	VAC072	Unvaccinated UK adult CHMI control
S4	C+35	Serum	VAC072	Unvaccinated UK adult CHMI control
S5	D84 (C-1)	Serum	VAC072	UK adult R21/MM 0,1,2 months (10,10,10ug)
S6	D84 (C-1)	Serum	VAC072	UK adult R21/MM 0,1,2 months (10,10,10ug)
S7	RC-1	Serum	VAC072	UK adult R21/MM 0,1,6 months (10,10,10ug)
S8	RC-1	Plasma	VAC072	UK adult R21/MM 0,1,6 months (10,10,2ug)
S9	C-1	Plasma	VAC072	Unvaccinated UK adult CHMI control
S10	C+35	Plasma	VAC072	Unvaccinated UK adult CHMI control
S11	C-1	Plasma	VAC072	Unvaccinated UK adult CHMI control
S12	C+35	Plasma	VAC072	Unvaccinated UK adult CHMI control
S13	RC-1	Plasma	VAC072	UK adult R21/MM 0,1,6 months (50,50,10 ug)
S14	C-1	Plasma	VAC072	UK adult R21/MM 0,1,2 months (10,10,10ug)
S15	RC-1	Plasma	VAC072	UK adult R21/MM 0,1,6 months (10,10,2ug)

CHMI – controlled human malaria infection, C+35 – Sporozoite challenge plus 35 days for CHMI controls (did not receive R21 vaccination), C-1 – Day prior to sporozoite challenge (1 month after 3 doses of R21/MM), RC-1 – Day prior to sporozoite rechallenge (between 5-7 months after previous sporozoite challenge). Adult – 18-45 years

Supplementary Note 2

Comparison of BSA-conjugated and native peptides

Two batches of 18 titration plates prepared each with 0.5x, 1x and 2x antigen concentrations

- Batch 1: Native, unconjugated peptides
- Batch 2: BSA-peptide conjugates

- Both batches of titration plates were also coated with three concentrations (100, 200 and 400 µg/mL) of R21 and HBsAg proteins
- BSA was coated on remaining spots (2, 4, 5, 6, 7 and 9)
- All coating solutions were prepared in dPBS with stabilizer

Supplementary Table 2 – Native Peptides

Plate	Spot	Antigen	µg/mL
0.5x	1	R21	100
	3	HBsAg	100
	8	CSP NANP	10
	10	CSP C-term	10
1x	1	R21	200
	3	HBsAg	200
	8	CSP NANP	20
	10	CSP C-term	20
2x	1	R21	400
	3	HBsAg	400
	8	CSP NANP	40
	10	CSP C-term	40

Supplementary Table 3 - BSA-Peptides (Plate Lot: R410614A)

Plate	Spot	Antigen	µg/mL
0.5x	1	R21	100
	3	HBsAg	100
	8	BSA-CSP NANP	750
	10	BSA-CSP C-term	750
1x	1	R21	200
	3	HBsAg	200
	8	BSA-CSP NANP	750
	10	BSA-CSP C-term	750
2x	1	R21	400
	3	HBsAg	400
	8	BSA-CSP NANP	750

10	BSA-CSP C-term	750

Determine an optimal dilution of human reference serum sample for plate uniformity testing

- The standard serology assay protocol was followed using 1x titration plates
- The human reference standard serum sample was diluted in Diluent 100 and tested at 100, 400, 1600, 6400, 25600, 102400 and 409600-fold dilutions in duplicate wells
- Diluent 100 (no sample) control was run to assess assay background
- Tested 10 plasma and 10 serum samples at 4 dilutions for each sample
    - 100, 1,000, 10,000 and 100,000-fold dilutions
- SULFO-TAG™ labeled anti-human IgG detection was used at 1 µg/mL

Results:

- Higher signals were observed with BSA-CSP peptide antigens than with native CSP peptides
- Selected 100,000-fold dilution for plate uniformity testing
- Selected 10,000-fold dilution for the standard curve top of curve (TOC)

Supplementary Table 4 - Native Peptides: Reference Standard Signals and Signal CVs

Native Peptides		Avg Signal (n=2)					Signal CV (n=2)				
DF	Ref Std	R21	HBsAg	CSP NANP	CSP C-term	BSA	R21	HBsAg	CSP NANP	CSP C-term	BSA
100	Std 1	8394538	6180629	751407	3419423	7192	1.3	1.2	5.7	3.5	91.1
400	Std 2	8525132	4669227	471144	2252316	1656	4.9	2.6	5.2	4.3	40.3
1600	Std 3	7160040	1929452	173944	853087	453	1.3	5.7	2.8	10.8	27.0
6400	Std 4	3506395	619572	46878	246469	56	2.5	3.6	2.7	0.3	121.7
25600	Std 5	960537	168889	11415	67099	85	4.1	3.7	1.3	2.8	72.4
102400	Std 6	262781	44181	3028	16799	84	0.2	3.3	1.1	7.7	74.3
409600	Std 7	72511	11795	865	4411	60	1.4	4.1	11.3	2.1	94.3
Diluent 100	Std 8	-42	-7	81	-6	-10	-158.5	-747.5	85.6	-330.0	-421.1

Supplementary Table 5 - BSA Conjugated Peptides: Reference Standard Signals and Signal CVs

BSA-Peptides		Avg Signal (n=2)					Signal CV (n=2)				
DF	Ref Std	R21	HBsAg	CSP NANP	CSP C-term	BSA	R21	HBsAg	CSP NANP	CSP C-term	BSA
100	Std 1	7658818	6242422	7550595	7003126	7157	2.6	1.1	6.0	0.1	47.9
400	Std 2	7534958	4395703	7559713	5928386	3331	1.7	5.6	0.5	1.9	55.8
1600	Std 3	6694085	1832752	5769757	2087547	1133	1.1	3.5	2.7	1.4	70.7
6400	Std 4	2581570	572835	1658975	589296	249	2.7	2.7	5.8	1.4	90.5

25600	Std 5	694440	158125	428109	158561	170	2.7	1.2	1.1	0.6	62.1
102400	Std 6	187784	42720	109339	39929	150	1.4	1.6	0.3	0.1	75.4
409600	Std 7	48848	11181	28831	10448	118	2.4	4.5	0.6	4.1	83.6
Diluent 100	Std 8	-3	-25	41	-21	18	-2064.8	-228.0	155.2	-182.8	265.5

Supplementary Figure 1 - BSA-conjugated peptides using plasma samples. Dotted lines at 1,000,000 and 300 signal represent high and low assay limits, respectively. 1,000-fold dilution is optimal for D0 and D35 samples, ≥100,000-fold dilution is optimal for D84 and D196 samples

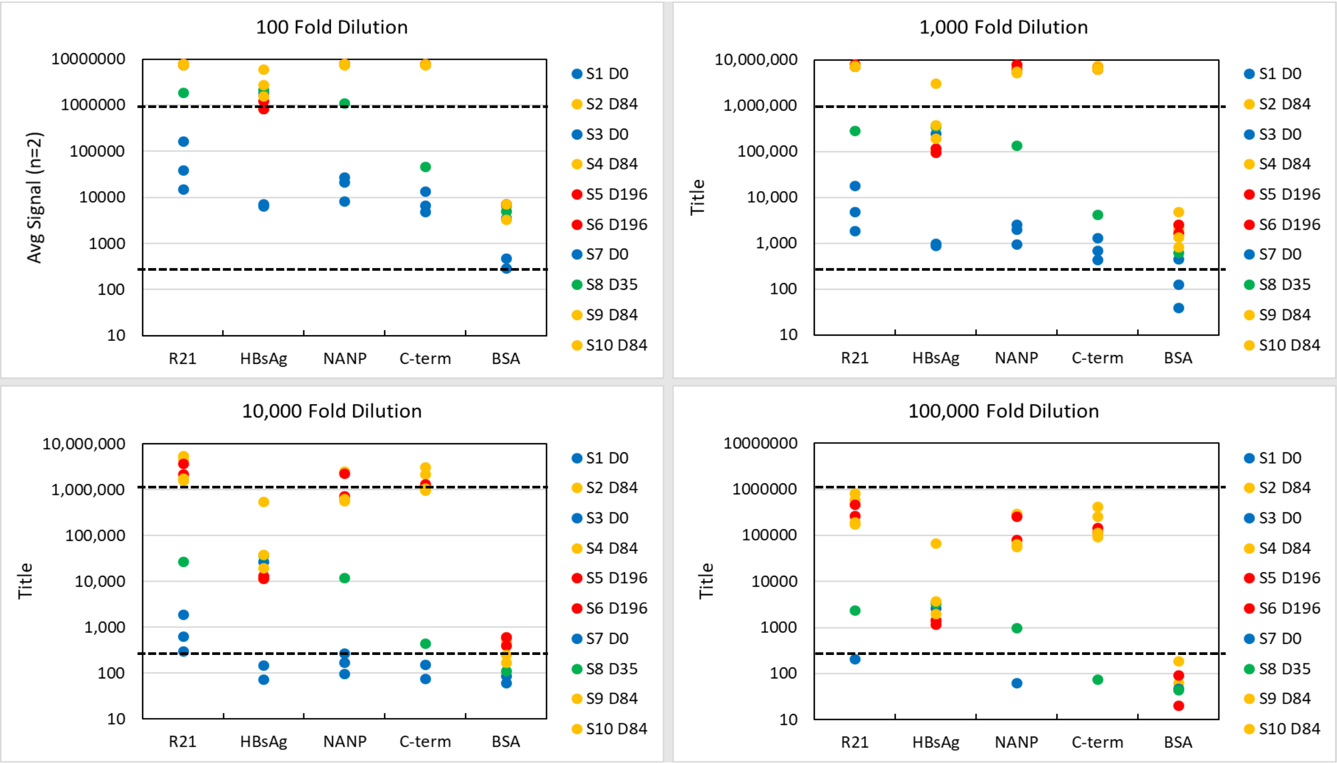

Supplementary Figure 2 - BSA-conjugated peptides using serum samples. Dotted lines at 1,000,000 and 300 signal represent high and low assay limits, respectively. 1,000-fold dilution is optimal for D0 and D35 samples, ≥100,000-fold dilution is optimal for D84 and D196 samples

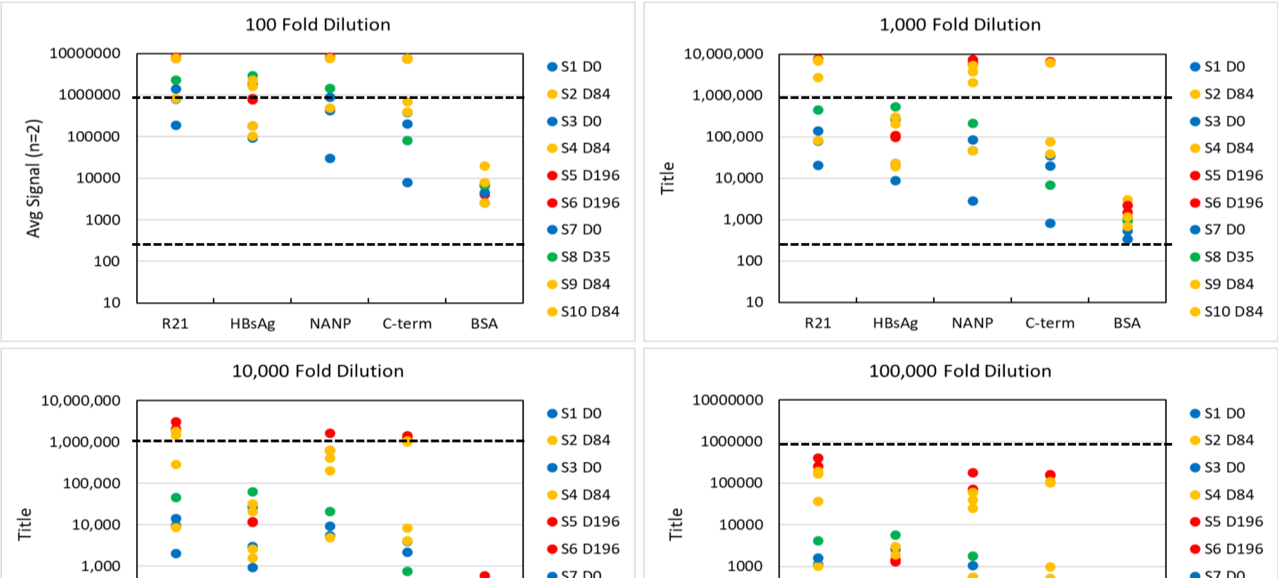

Conclusions:

- Wide range of signals across the four study time points (D0, D35, D84 and D196)
- Multiple sample dilution factors will be required in order to measure the full range of titers
  - 1,000-fold for D0 and D35
  - 10,000, and 100,000-fold for D84 and D196
- BSA-peptide signals for CSP NANP and CSP C-terminus are higher than the native, unconjugated peptides

Assess coating uniformity and signal at each antigen concentration using a single reference sample dilution

- The standard serology assay protocol was followed using 1x titration plates
- The human reference standard serum sample was diluted in Diluent 100 and tested at 100,000-fold dilution across the entire plate
- Two plates from each of 6 titration plate lots were tested
  - Native peptide plates: 0.5x, 1x and 2x
  - BSA-peptide plates: 0.5x, 1x and 2x
- SULFO-TAG™ labeled anti-human IgG detection was used at 1 µg/mL

Results:

- Native peptide plates:
  - HBsAg, CSP NANP and CSP C-terminus signals increase with increasing concentration
  - R21 signals remain constant on the native peptide plates
- BSA-peptide plates:
  - HBsAg signals increase with increasing concentration
  - BSA-CSP NANP and BSA-CSP C-Terminus signals plateau between 1x and 2x
  - R21 signals decrease with increasing concentration

Supplementary Table 6. Native vs BSA-conjugated peptides

	Native Peptide Plates											
	Avg Intraplate Signal (n=192)			Normalized Signal			Avg Intraplate CV			Max Intraplate CV		
Assay	0.5x	1x	2x	0.5x	1x	2x	0.5x	1x	2x	0.5x	1x	2x
R21	246,016	246,121	250,959	98%	98%	100%	3.5%	3.3%	3.4%	3.8%	4.1%	3.6%
HBsAg	17,605	44,818	68,056	26%	66%	100%	8.3%	5.0%	5.1%	11.3%	5.7%	6.3%
CSP NANP	1,217	3,417	7,862	15%	43%	100%	10.8%	9.0%	7.5%	12.5%	9.1%	8.6%
CSP C-term	11,981	19,659	27,393	44%	72%	100%	7.2%	7.6%	7.2%	7.9%	9.2%	7.4%

	BSA-Peptide Plates											
	Avg Intraplate Signal (n=192)			Normalized Signal			Avg Intraplate CV			Max Intraplate CV		
Assay	0.5X	1X	2X	0.5X	1X	2X	0.5X	1X	2X	0.5x	1x	2x
R21	205,125	173,815	154,945	132%	112%	100%	4.3%	4.4%	5.4%	4.3%	5.1%	5.7%
HBsAg	18,094	42,199	70,465	26%	60%	100%	8.9%	4.4%	5.1%	10.5%	5.1%	5.4%
BSA-CSP NANP	73,325	97,748	103,325	71%	95%	100%	5.7%	4.1%	5.4%	6.1%	4.8%	6.7%
BSA-CSP C-term	24,212	40,201	49,501	49%	81%	100%	2.8%	2.2%	3.5%	3.0%	2.4%	4.8%

Supplementary Figure 3 - Determination of optimal coating concentration

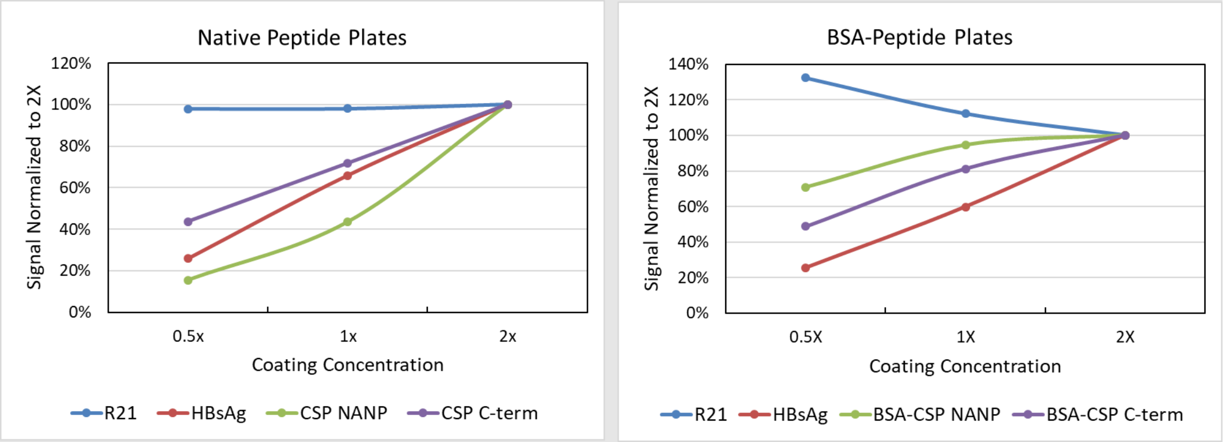

- Signals for the BSA-peptides are higher and plateau while signals for the native peptides do not plateau
- Intraplate %CVs are typically <10% and are lower for the BSA-peptide plates than for the native peptide plates

- Although R21 is coated at the same concentration across batches of plates, R21 signals are constant across coating concentrations in the first batch and decrease with increasing concentration in the second batch of titration plates

Conclusions:

- Signals for BSA-peptide antigens BSA-CSP NANP and BSA-CSP C-terminus are higher than the signals of the native peptides and plateau between 1x and 2x
- Signals for the native peptides increase and do not reach a plateau
- Intraplate %CVs are lower for the BSA-peptide antigens than the native peptides
- Based on signals and CVs, MSD recommends coating plate with BSA-conjugated CSP NANP and CSP C-terminal peptides
- Preliminary antigen coating concentrations for production plates
    - R21: 0.5x (100 µg/mL)
    - HBsAg: 2x (400 µg/mL)
    - BSA-CSP NANP: 1x
    - BSA-CSP C-terminus: 1x

Assess coating uniformity and signal of each antigen at a single reference sample dilution

- The standard serology assay protocol was followed using 1x titration plates
- The human reference standard serum sample was diluted in Diluent 100 and tested at 100,000-fold dilution across the entire plate
- Three plates from the production plate lot and each concentration (0.5x, 1x and 2x) of titration plate lot (with BSA-peptides) were tested
- SULFO-TAG™ labeled anti-human IgG detection was used at 1 µg/mL

Results:

- Low intraplate CVs (<10%) for each antigen were observed
- Signals and CVs similar to titration plates were observed

Supplementary Table 7 - Summary of coating uniformity

Production Plates	Antigen			
	R21	HBsAg	CSP NANP	CSP C-terminus
Average Signal (n=3)	212649	88856	103937	45103
CV of Intraplate Averages (n=3)	3.0%	3.3%	5.8%	2.7%
Average intraplate CV	4.0%	5.1%	4.8%	3.1%
Max intraplate CV	4.8%	6.4%	6.1%	3.8%

Titration Plates	Antigen			

	R21 (0.5x)	HBsAg (2x)	CSP NANP (1x)	CSP C-terminus (1x)
Average Signal (n=3)	222987	73662	104610	42872
CV of Intraplate Averages (n=3)	4.2%	1.9%	5.2%	4.9%
Average intraplate CV	4.3%	4.5%	4.1%	2.5%
Max intraplate CV	5.7%	4.9%	5.0%	3.3%

Conclusions:

- Good plate uniformity (<10% intraplate CV) was observed for all assays

Assess signals and calculated concentrations of serum and plasma samples at two dilutions for each sample and Comparison of results from the production plates to the titration plates

- The standard serology assay protocol was followed using the production plate lot and each of the three concentrations of the BSA-peptide titration plate lot for four study time points (D0, D35, D84 and D196)
- An 8 point standard curve was made from the human reference standard serum sample diluted in Diluent 100 to 10,000-fold top of curve (TOC) and serial diluted in 4-fold steps
- 20 serum and 20 plasma samples were tested with duplicate replicates
    - 5 samples from each of the 4 study time points were tested at two dilutions
    - 1000 and 10,000-fold for D0 and D35
    - 100,000 and 1,000,000-fold for D84 and D196
- SULFO-TAG™ labeled anti-human IgG detection was used at 1 µg/mL

Results:

- Performance of production plates for all assays was similar to performance observed with titration plates

Supplementary Table 8 – Performance of production plates

			Production Plates		0.5x Titration Plates		
**Assay**	**Sample**	**AU/mL**	**Avg. Signal (n=4)**	**CV (%)**	**Avg. Signal (n=4)**	**CV (%)**	**Percent of 0.5x**
**R21**	Std 1	9.300	1762577	2.4	1974076	1.8	89
	Std 2	2.325	482765	1.7	537263	1.5	90
	Std 3	0.581	121491	4.6	139246	3.1	87
	Std 4	0.145	31688	2.1	34684	2.3	91
	Std 5	0.036	7969	3.8	9076	1.5	88
	Std 6	0.009	2107	1.7	2407	1.4	88
	Std 7	0.002	575	3.6	645	1.3	89
	Std 8	0.000	130	3.0	95	25.5	136

	**Hill Slope**	1.01	0.99	101
	**LLOD**	0.00037	0.00030	123

			**Production Plates**		**2x Titration Plates**		
**Assay**	**Sample**	**AU/mL**	**Avg. Signal (n=4)**	**CV (%)**	**Avg. Signal (n=4)**	**CV (%)**	**Percent of 2x**
**HBsAg**	Std 1	4.000	782670	1.2	618391	1.5	127
	Std 2	1.000	206351	2.5	168475	1.0	122
	Std 3	0.250	52223	3.5	41012	1.0	127
	Std 4	0.063	12701	9.8	10237	3.0	124
	Std 5	0.016	3479	3.2	2773	4.4	125
	Std 6	0.004	960	3.9	770	8.3	125
	Std 7	0.001	300	8.1	245	11.0	123
	Std 8	0.000	88	15.1	97	14.2	91
	**Hill Slope**		0.99		1.01		98
	**LLOD**		0.00034		0.00046		73

			**Production Plates**		**1x Titration Plates**		
**Assay**	**Sample**	**AU/mL**	**Avg. Signal (n=4)**	**CV (%)**	**Avg. Signal (n=4)**	**CV (%)**	**Percent of 0.5x**
**CSP NANP**	Std 1	6.400	998381	3.0	983497	1.7	102
	Std 2	1.600	243682	2.0	243684	1.4	100
	Std 3	0.400	62137	6.2	59654	3.6	104
	Std 4	0.100	15714	1.9	14824	3.5	106
	Std 5	0.025	3957	8.4	3875	2.5	102
	Std 6	0.006	1025	5.7	1010	5.3	101
	Std 7	0.002	316	11.3	282	2.7	112
	Std 8	0.000	105	4.4	77	14.2	137
	**Hill Slope**		1.01		1.02		100
	**LLOD**		0.00053		0.00055		97

			**Production Plates**		**1x Titration Plates**		
**Assay**	**Sample**	**AU/mL**	**Avg. Signal (n=4)**	**CV (%)**	**Avg. Signal (n=4)**	**CV (%)**	**Percent of 0.5x**
**CSP C-term**	Std 1	3.200	431689	2.8	410889	3.6	105
	Std 2	0.800	106579	1.3	102996	1.6	103
	Std 3	0.200	27205	1.0	26093	0.6	104
	Std 4	0.050	6689	0.4	6466	0.4	103
	Std 5	0.013	1838	2.8	1755	2.9	105
	Std 6	0.003	491	1.6	483	4.7	102
	Std 7	0.001	163	5.6	162	2.0	101
	Std 8	0.000	69	3.0	48	75.4	145
	**Hill Slope**		1.01		0.99		102
	**LLOD**		0.00057		0.00057		101

- Standard curve performance for plasma and serum plates are shown
- Performance of production plates for all assays was similar to performance observed with titration plates

Supplementary Figure 4 – Standard curve performance

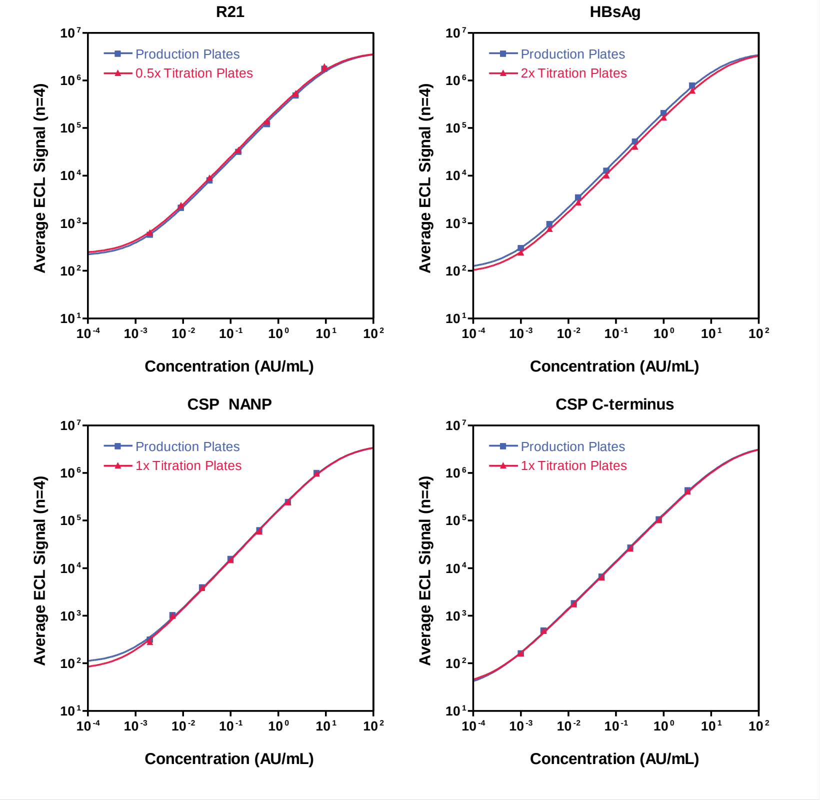

- Reference standard serum sample was diluted 10,000-fold (standard 1) and serial diluted 4-fold to make an 8 point standard curve
- Arbitrary units per mL (AU/mL) were assigned for each antigen as described previously
- Standard curves are shown for plasma and serum sample plates for each assay
- Standard curves for the production plates are similar to the titration plates

Supplementary Figure 5 – Plasma and serum tested on titration and production plates

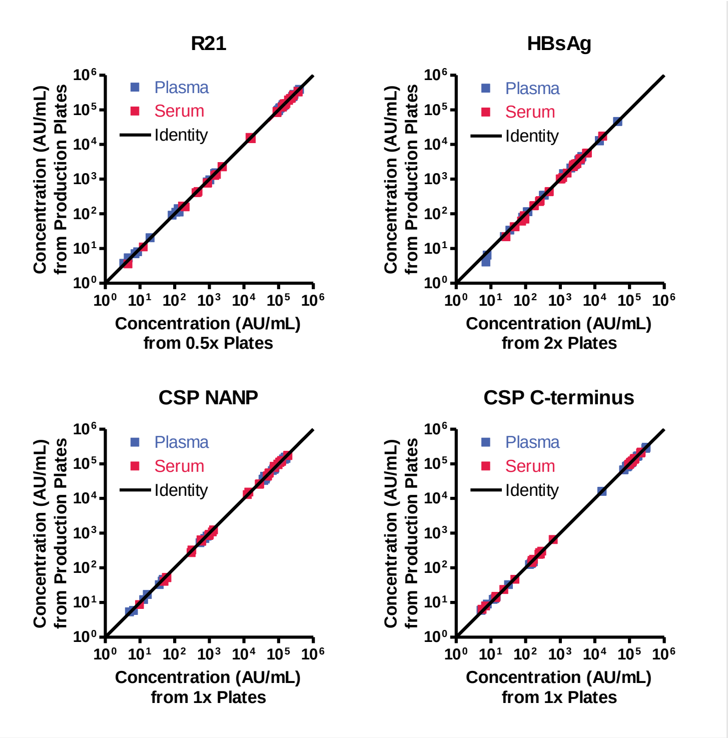

- Antigen concentrations (AU/mL) from production plates were compared to titration plates
- Concentrations measured from the production plates had a good correlation with concentrations measured from titration plates

Supplementary Table 9: Summary of slopes

Antigen	Test	Parameter	Formatting	Production Plates
R21	Uniformity (n=3 plates)	Avg. Signal	1,500-1,000,000	212,649
		CV of Intraplate Averages	<18%	3.0
		Avg. Intraplate CV (%)	<10%	4.0
		Max. Intraplate CV (%)	<10%	4.8
	Functional Plasma	n	NA	35
		Slope	0.8 - 1.2	0.97
		Median % Difference	-20% - 20%	1.2
	Functional Serum	n	NA	36
		Slope	0.8 - 1.2	0.94
		Median % Difference	-20% - 20%	6.3
HBsAg	Uniformity (n=3 plates)	Avg. Signal	1,500-1,000,000	88,856
		CV of Intraplate Averages	<18%	3.3
		Avg. Intraplate CV (%)	<10%	5.1
		Max. Intraplate CV (%)	<10%	6.4

	Functional Plasma	n<br>Slope<br>Median % Difference	NA<br>0.8 - 1.2<br>-20% - 20%	34<br>1.01<br>1.3
	Functional Serum	n<br>Slope<br>Median % Difference	NA<br>0.8 - 1.2<br>-20% - 20%	34<br>1.04<br>4.6
**BSA-CSP NANP**	Uniformity (n=3 plates)	Avg. Signal<br>CV of Intraplate Averages<br>Avg. Intraplate CV (%)<br>Max. Intraplate CV (%)	1,500-1,000,000<br><18%<br><10%<br><10%	103,937<br>5.8<br>4.8<br>6.1
	Functional Plasma	n<br>Slope<br>Median % Difference	NA<br>0.8 - 1.2<br>-20% - 20%	34<br>0.95<br>3.3
	Functional Serum	n<br>Slope<br>Median % Difference	NA<br>0.8 - 1.2<br>-20% - 20%	36<br>0.98<br>3.4
**BSA-CSP C-term**	Uniformity (n=3 plates)	Avg. Signal<br>CV of Intraplate Averages<br>Avg. Intraplate CV (%)<br>Max. Intraplate CV (%)	1,500-1,000,000<br><18%<br><10%<br><10%	45,103<br>2.7<br>3.1<br>3.8
	Functional Plasma	n<br>Slope<br>Median % Difference	NA<br>0.8 - 1.2<br>-20% - 20%	37<br>0.98<br>0.99
	Functional Serum	n<br>Slope<br>Median % Difference	NA<br>0.8 - 1.2<br>-20% - 20%	36<br>1.00<br>-0.58

Supplementary Table 10: sample testing data

**Plasma**				**Avg. Signal (n=2)**		**Dill. Corr. AU/mL**		**Conc. CV (%)**		
**PID**	**TP**	**DF**	**ID**	**0.5x**	**Production**	**0.5x**	**Production**	**0.5x**	**Production**	**Percent of 0.5x**
43	D0	1000	U001	21689	30637	86.9	150.3	1.6	55.2	173
77	D0	1000	U002	1274	1225	4.6	5.4	6.0	13.9	117
78	D0	1000	U003	975	909	3.4	3.8	2.9	0.9	112
120	D0	1000	U004	5028	4279	19.6	20.6	1.6	0.4	105
408	D0	1000	U005	1928	1564	7.2	7.1	2.2	1.5	98
43	D0	10000	U006	2231	1978	84.4	91.8	1.1	5.3	109
77	D0	10000	U007	232	224	4.9	4.0	22.9	61.3	82
78	D0	10000	U008	201	168	3.7	1.1	41.1	29.4	30
120	D0	10000	U009	602	652	19.5	25.6	1.9	13.1	131
408	D0	10000	U010	319	275	8.3	6.6	23.5	18.5	79
43	D35	1000	U011	370835	312911	1540	1544	0.5	9.2	100
77	D35	1000	U012	33706	22991	136	113	0.7	4.8	83
78	D35	1000	U013	2275	1759	8.6	8.1	5.0	4.9	94

Plasma				Avg. Signal (n=2)		Dill. Corr. AU/mL		Conc. CV (%)		
PID	TP	DF	ID	0.5x	Production	0.5x	Production	0.5x	Production	Percent of 0.5x
80	D35	1000	U014	34138	53374	137	262	1.1	65.5	191
83	D35	1000	U015	251849	195111	1037	959	0.1	4.5	92
43	D35	10000	U016	35299	30057	1421	1475	0.8	4.3	104
77	D35	10000	U017	2820	2374	108	112	2.8	16.5	103
78	D35	10000	U018	292	304	7.2	8.0	30.8	13.8	111
80	D35	10000	U019	3263	2988	126	142	1.0	1.8	113
83	D35	10000	U020	21977	17005	880	834	1.9	6.3	95
37	D84	100000	U021	231387	195976	95161	96290	0.2	6.7	101
44	D84	100000	U022	230368	190622	94735	93644	0.9	2.0	99
54	D84	100000	U023	233572	192044	96076	94346	2.9	1.2	98
120	D84	100000	U024	631822	499572	266783	248353	2.9	0.2	93
408	D84	100000	U025	848013	693168	362795	347774	1.0	0.3	96
37	D84	1000000	U026	24954	19684	100076	96541	0.5	8.5	96
44	D84	1000000	U027	24648	19908	98837	97642	1.0	2.2	99
54	D84	1000000	U028	24988	20440	100216	100259	0.2	1.2	100
120	D84	1000000	U029	69174	57495	280176	282015	1.3	1.4	101
408	D84	1000000	U030	97940	80032	398115	392495	0.6	0.5	99
8	D196	100000	U031	601951	478045	253699	237421	0.6	1.4	94
16	D196	100000	U032	326929	266487	135374	131222	3.5	2.4	97
34	D196	100000	U033	310419	256448	128390	126236	1.3	4.0	98
42	D196	100000	U034	330605	268360	136929	132153	1.4	2.8	97
75	D196	100000	U035	260475	226947	107354	111607	2.0	3.4	104
8	D196	1000000	U036	63244	50334	255931	246911	1.3	2.7	96
16	D196	1000000	U037	35085	28155	141186	138146	1.2	0.5	98
34	D196	1000000	U038	33172	28705	133416	140845	1.4	4.9	106
42	D196	1000000	U039	34566	28475	139076	139714	2.8	3.5	100
75	D196	1000000	U040	27647	23540	110995	115488	1.8	5.5	104

Conclusions:

- Good uniformity and correlation with titration plates
- Multiple sample dilution factors are required to cover the full range of sample titers:
    - 1000-fold for D0 and D35
    - 10,000 and 100,000-fold or higher for D84 and D196
- Next Steps:
- Assay specificity testing
- Concentration assignment of HBsAg using WHO NIBSC standard 07/164
- Protocol optimization testing
    - Blocking time and conditions
    - Sample incubation time
    - Detection incubation time

Assessing the specificity of each assay

- Specific and non-specific signals were assessed using antibodies specific for each antigen
- The standard serology assay protocol was followed using the production plate lot (R410715A))
- An 8 point standard curve with 4-fold steps was made from the following:
  - Human reference standard serum sample (diluted 10,000-fold to Top Of Curve (TOC))
  - Anti-HB human immunoglobulin (diluted to 0.05 IU/mL TOC)
    - WHO International Standard NIBSC 07/164
  - Anti-CSP NANP mouse monoclonal antibody (diluted to 5 µg/mL TOC)
  - Anti-CSP C-terminus mouse monoclonal antibody (diluted to 5 µg/mL TOC)
- SULFO-TAG™ labeled anti-human IgG detection (HyTest 3D3cc) was used at 1 µg/mL
- SULFO-TAG™ labeled anti-mouse IgG detection (Thermo 31232) was used at 4 µg/mL
- Percent non-specific binding (NSB) was calculated as:
  - % NSB = 100 x signal - background / specific signal - background

Results:

- The R21 assay shows expected cross-reactivity with each antigen
- The HBsAg, CSP NANP and CSP C-terminus assays show good specificity (%NSB <1%)
- Signals at a single level of reference standard or antibody are shown (specific signals are shaded grey)
- Percent non-specific binding was calculated as:
    - % NSB = 100 x signal - background / specific signal – background
- As expected, each antibody shows cross-reactivity with the R21 antigen
- The HBsAg, CSP NANP and CSP C-terminus assays show good specificity (%NSB <1%)

Supplementary Table 11 - Non-specific binding

**NSB (%)**		**Antigen**			
		**R21**	**HBsAg**	**CSP NANP**	**CSP C-term**
**Antibody**	**Ref Std**	100	100	100	100
	**anti-HB**	8.3	100	0.48	0.28
	**anti-CSP NANP**	77	-0.03	100	0.09
	**anti-CSP C-term**	105	0.34	0.10	100

**Signals**		**Antigen**			
		**R21**	**HBsAg**	**CSP NANP**	**CSP C-term**
	**Ref Std**	111174	46534	56396	24435

**Antibody**	**anti-HB**	8789	104814	567	335
	**anti-CSP NANP**	49401	114	64352	604
	**anti-CSP C-term**	22194	166	158	21403

**Background Signals**		**Antigen**			
		**R21**	**HB**	**CSP NANP**	**CSP C-term**
**Antibody**	**Ref Std**	105	58	66	16
	**anti-HB**	45	43	67	36
	**anti-CSP NANP**	236	132	151	548
	**anti-CSP C-term**	196	96	138	534

Concentration Assignment of HBsAg

Assign a concentration to HBsAg in the human reference standard serum sample using the WHO NIBSC standard 07/164

- WHO NIBSC standard 07/164 was prepared according to the CoA
    - Material in a single glass ampule was reconstituted to 100 IU/mL with 1 mL of water
    - The 100 IU/mL NIBSC standard stock was diluted to 1 IU/mL with Diluent 100
    - The NIBSC standard was further diluted 20-fold with Diluent 100 to 0.05 IU/mL
    - The human reference serum sample was diluted 10,000-fold with Diluent 100
- 8 point standard curves were made from the NIBSC standard (starting at 0.05 IU/mL) and human reference standard serum sample starting at a 10,000-fold dilution and serial diluted in 4-fold steps in six replicate wells for each standard at each dilution
- SULFO-TAG™ labeled anti-human IgG detection was used at 1 µg/mL
- The concentration of HBsAg in the human reference standard serum sample was measured by back-fitting signals from the human reference standard serum sample to the HB standard curve

Results:

- The HBsAg concentration in the human reference standard serum sample stock was assigned 243 IU/mL

Supplementary Table 12 – Assigning International Unit (IU) concentration for HBsAg

anti-HB	IU/mL	Avg Signal (n=6)	CV (%)
Std 1	0.050000	1697809	3.2
Std 2	0.012500	459058	2.9
Std 3	0.003125	111616	1.5
Std 4	0.000781	29425	1.4
Std 5	0.000195	7866	3.5
Std 6	0.000049	2182	3.1
Std 7	0.000012	545	6.6
Std 8	0.000000	89	27.1

Hill slope	0.99
LLOD (IU/mL)	0.00000181

Ref Std	DF	Avg Signal (n=6)	CV (%)	Dilution Corrected (IU/mL)
Std 1	10000	795819	3.0	225
Std 2	40000	216980	2.8	237
Std 3	160000	57756	2.5	246
Std 4	640000	14870	2.7	247
Std 5	2560000	4062	2.3	260
Std 6	10240000	1078	2.8	254
Std 7	40960000	312	12.1	225
Std 8	No Sample	72	29.2	0

	Assigned IU/mL
Avg. Std 2-4	243

- A stock of anti-HB WHO NIBSC Standard (07/164) at 1 IU/mL was diluted 20-fold to 0.05 IU/mL TOC and serial diluted with 4-fold steps to make an 8 point standard curve
- The human reference standard sample was diluted 10,000-fold TOC and serial diluted with 4-fold steps
- HBsAg concentration in the human reference standard sample was assigned as 243 IU/mL by averaging the dilution corrected concentration of Std 2-4 values (shaded green)
- Std points values used for concentration assignment were selected based on signals falling within the linear part of the ant-HB standard curve
- A concentration of 243 IU/mL for HBsAg in the human reference standard sample will be used in future tests
- The TOC for HBsAg will be 0.0243 IU/mL after 10,000-fold dilution of the human reference standard sample

Evaluate protocol incubation times and blocking conditions

- The standard serology assay protocol was followed using the production plate lot
- Incubation times shorter and longer than the standard protocol will be assessed for each step as follows:
  - Blocking at 15, 30, and 60 minutes
  - Blocking for 30 minutes with Diluent 100 will be compared to Blocker A
  - Sample incubation at 1, 2 and 3 hours
  - Detection incubation at 30 minutes, 1 hour and 2 hours
- An 8 point standard curve was made from the human reference standard serum sample diluted in Diluent 100 to 10,000-fold top of curve (TOC) and serial diluted in 4-fold steps

- Samples were pre-diluted and frozen in multiwell plates for use in subsequent protocol optimization tests
- 10 serum and 10 plasma samples were tested with duplicate replicates
  - 5 samples from each of the 4 study time points were tested at two dilutions
  - 1000 and 10,000-fold for D0 and D35
  - 100,000 and 1,000,000-fold for D84 and D196
- SULFO-TAG™ labeled anti-human IgG detection was used at 1 µg/mL

Results:

- Standard serology protocol times and conditions are recommended
- Good sample correlation for each condition
- Standard protocol incubation times and conditions are recommended

Supplementary Table 13 - Summary of Blocking Time and Conditions Optimization

Antigen	Parameter	Formatting	15 min	30 min Dill 100	60 min
**R21**	n	NA	34	37	37
	Slope	0.8 - 1.2	1.04	1.02	1.07
	Median % Difference	-20% - 20%	3.05	0.19	8.69
**HBsAg**	n	NA	35	34	35
	Slope	0.8 - 1.2	1.02	1.01	1.02
	Median % Difference	-20% - 20%	-1.81	-0.50	0.51
**BSA-CSP NANP**	n	NA	33	37	36
	Slope	0.8 - 1.2	1.07	1.01	1.02
	Median % Difference	-20% - 20%	5.61	3.02	8.31
**BSA-CSP C-term**	n	NA	38	38	38
	Slope	0.8 - 1.2	1.01	1.00	1.02
	Median % Difference	-20% - 20%	3.29	-0.35	0.84

Supplementary Table 14 - Summary of Sample Incubation Time Optimization

Antigen	Parameter	Formatting	1 hour	3 hours
**R21**	n	NA	36	36
	Slope	0.8 - 1.2	0.99	0.98
	Median % Difference	-20% - 20%	-3.16	-2.06
**HBsAg**	n	NA	31	33
	Slope	0.8 - 1.2	0.95	1.03
	Median % Difference	-20% - 20%	-1.68	-3.04
**BSA-CSP NANP**	n	NA	34	34
	Slope	0.8 - 1.2	0.98	0.97
	Median % Difference	-20% - 20%	-1.36	-6.27

**BSA-CSP C-term**	n	NA	38	37
	Slope	0.8 - 1.2	1.04	0.99
	Median % Difference	-20% - 20%	2.50	-0.70

Supplementary Table 15 - Summary of Detection Incubation Time Optimization

Antigen	Parameter	Formatting	30 min	2 hours
**R21**	n	NA	35	35
	Slope	0.8 - 1.2	0.97	1.04
	Median % Difference	-20% - 20%	-0.89	5.34
**HBsAg**	n	NA	36	35
	Slope	0.8 - 1.2	0.99	1.00
	Median % Difference	-20% - 20%	3.20	1.56
**BSA-CSP NANP**	n	NA	36	37
	Slope	0.8 - 1.2	0.98	1.07
	Median % Difference	-20% - 20%	-0.23	3.94
**BSA-CSP C-term**	n	NA	36	37
	Slope	0.8 - 1.2	0.98	1.07
	Median % Difference	-20% - 20%	1.30	1.06

- Good sample correlation for each condition
- Standard protocol incubation times and conditions are recommended

Supplementary Figure 6 - Blocking Time and Conditions: % Difference

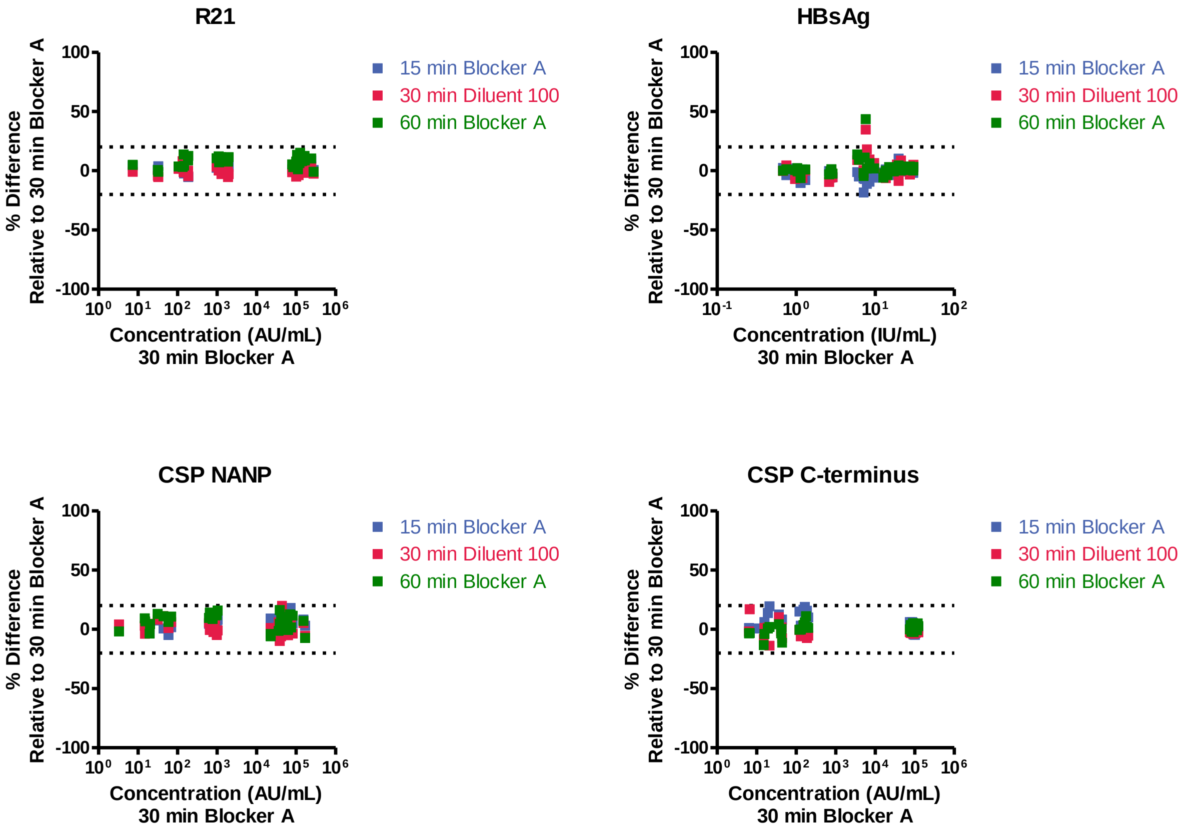

- Percent difference of sample concentrations relative to the standard blocking condition (30 minutes with
- Blocker A) were plotted
- Percent difference of sample concentrations are mostly within 20% (dotted lines)

Supplementary Figure 7 - Blocking Time and Conditions: Deming Plots

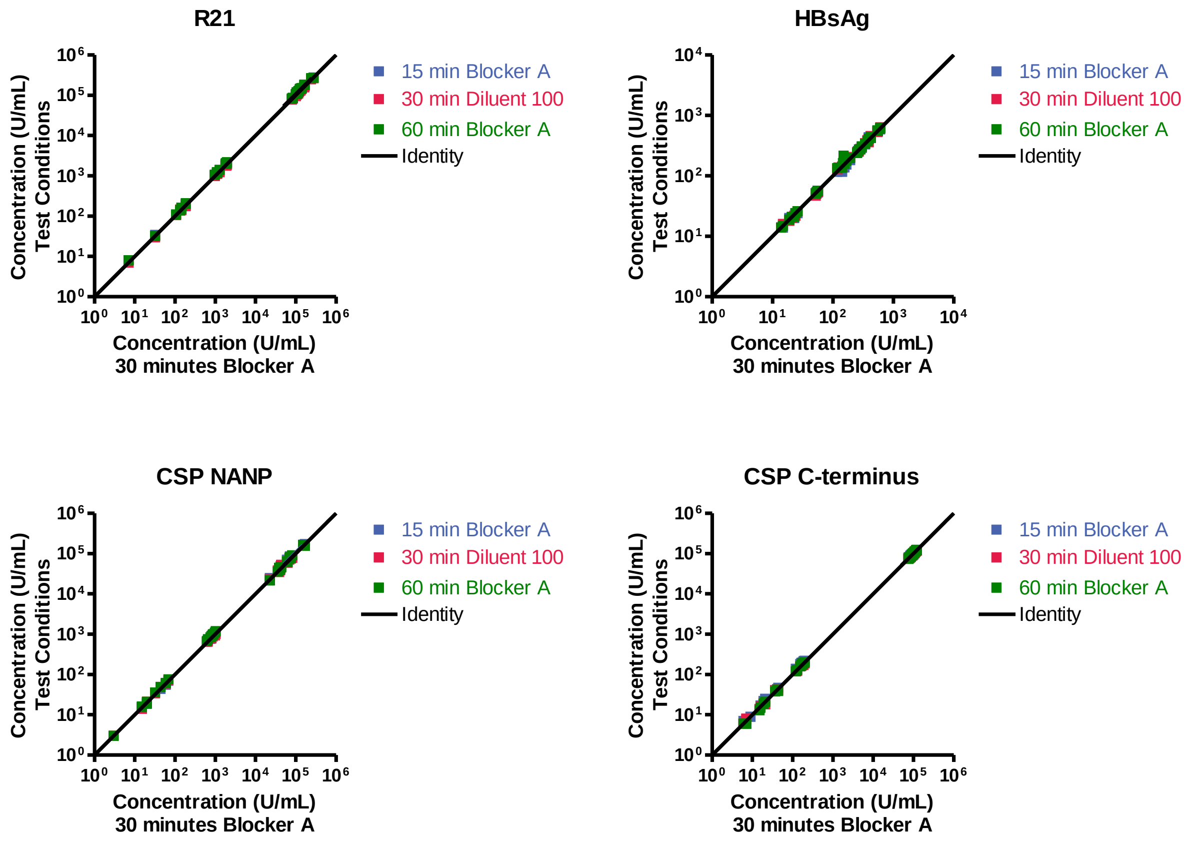

- Sample concentrations from different blocking times and conditions were compared to the standard condition (30 minutes with Blocker A)
- Good correlation of sample concentrations were observed with each condition

Supplementary Figure 8 - Sample Incubation Time: % Difference

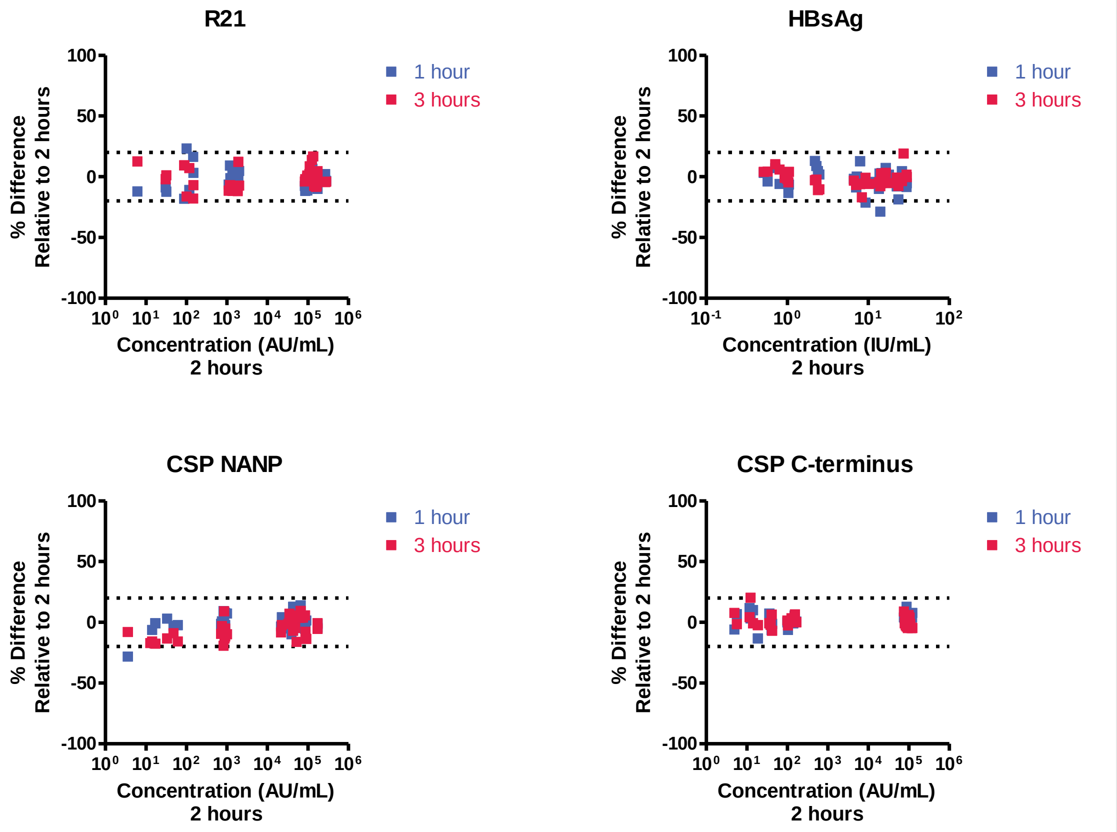

- Percent difference of sample concentrations relative to the standard incubation time (2 hours) were plotted
- Percent difference of sample concentrations are mostly within 20% (dotted lines)

Supplementary Figure 9 - Sample Incubation Time: Deming Plots

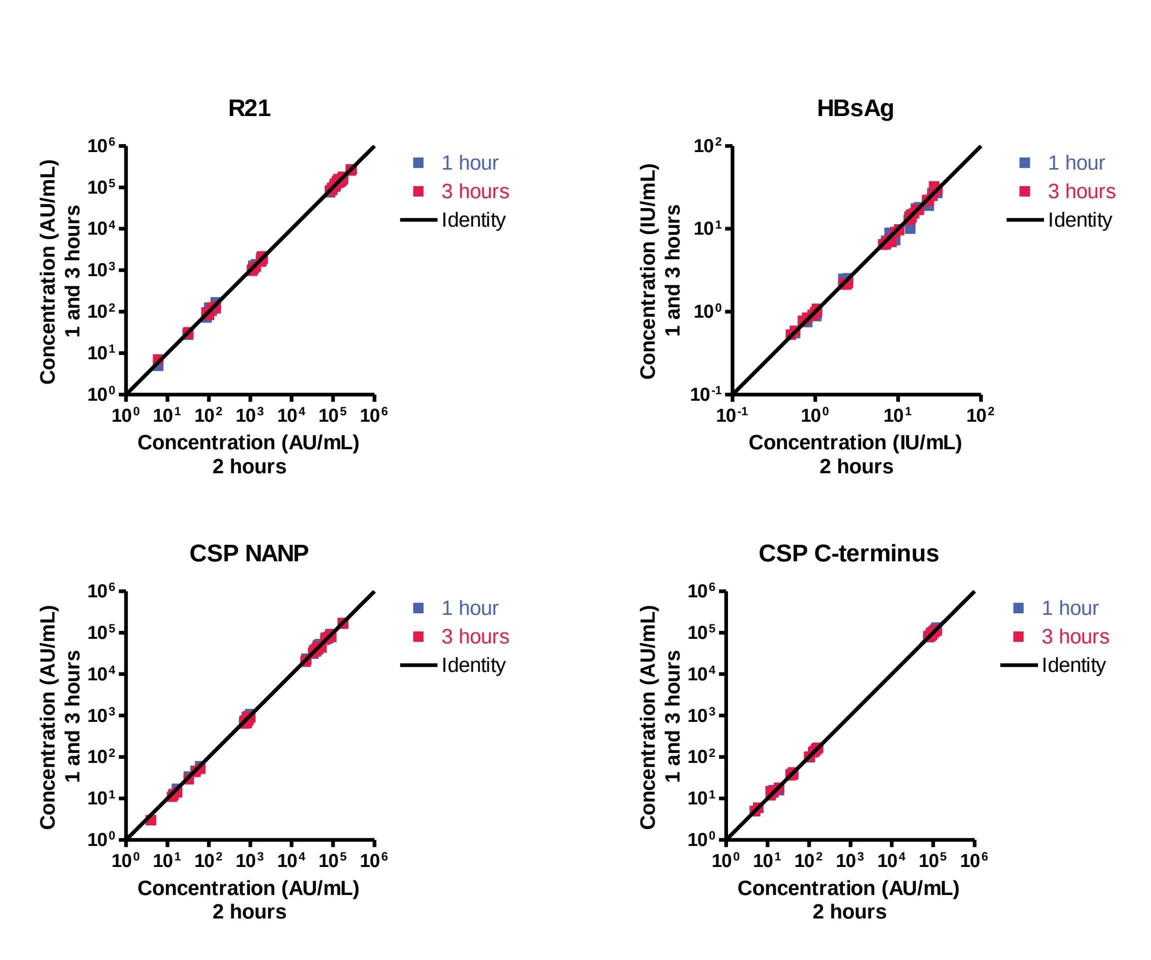

- Sample concentrations from 1 hour and 2 hour sample incubation times were compared to the standard condition (2 hours)
- Good correlations of sample concentrations were observed for each condition

Supplementary Figure 10 - Detection Incubation Time: % Difference

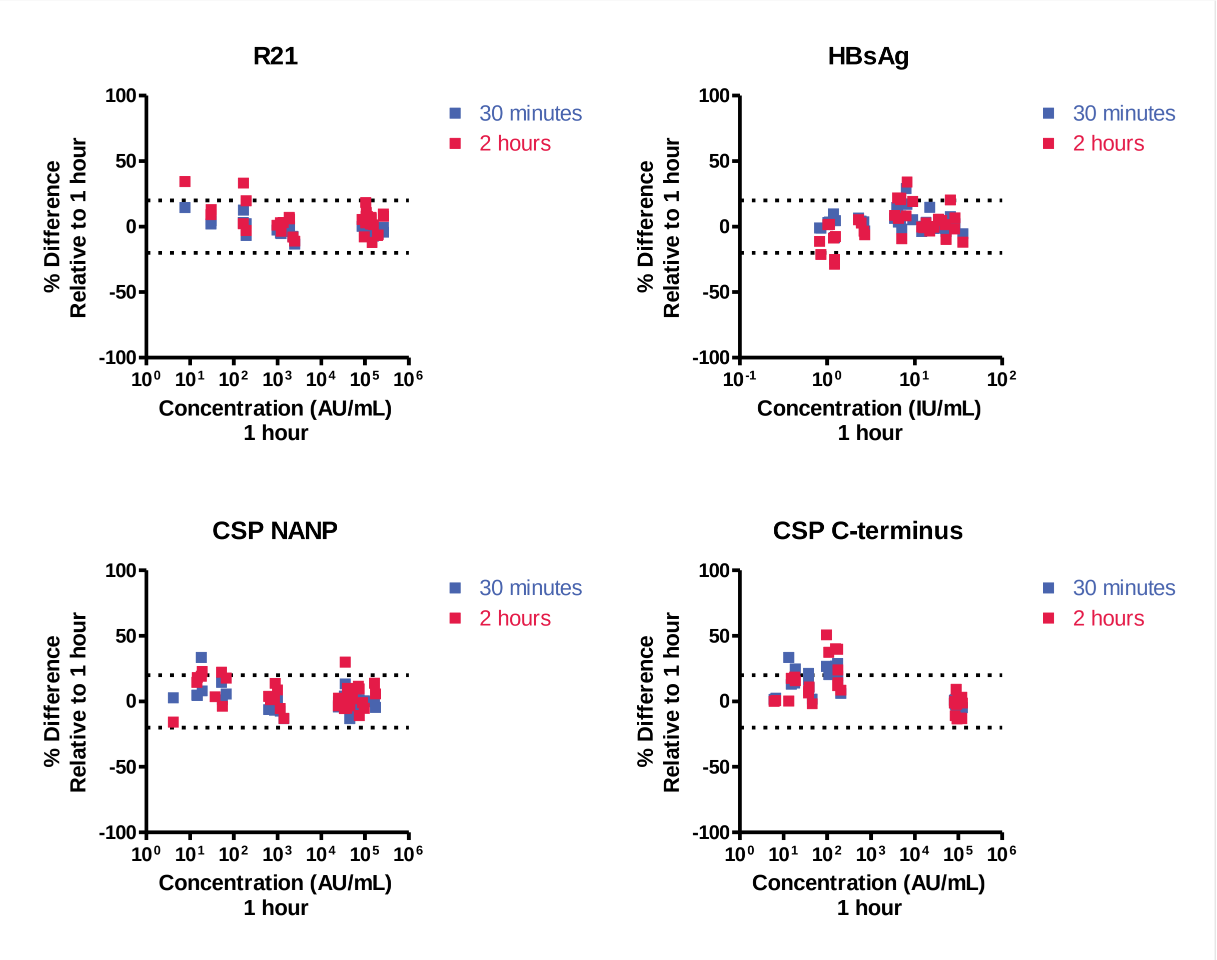

- Percent difference of sample concentrations relative to the standard incubation time (1 hours) were plotted
- Sample concentrations at 30 minutes and 2 hours are mostly within 20% (dotted lines)

Supplementary Figure 11 - Detection Incubation Time: Deming Plots

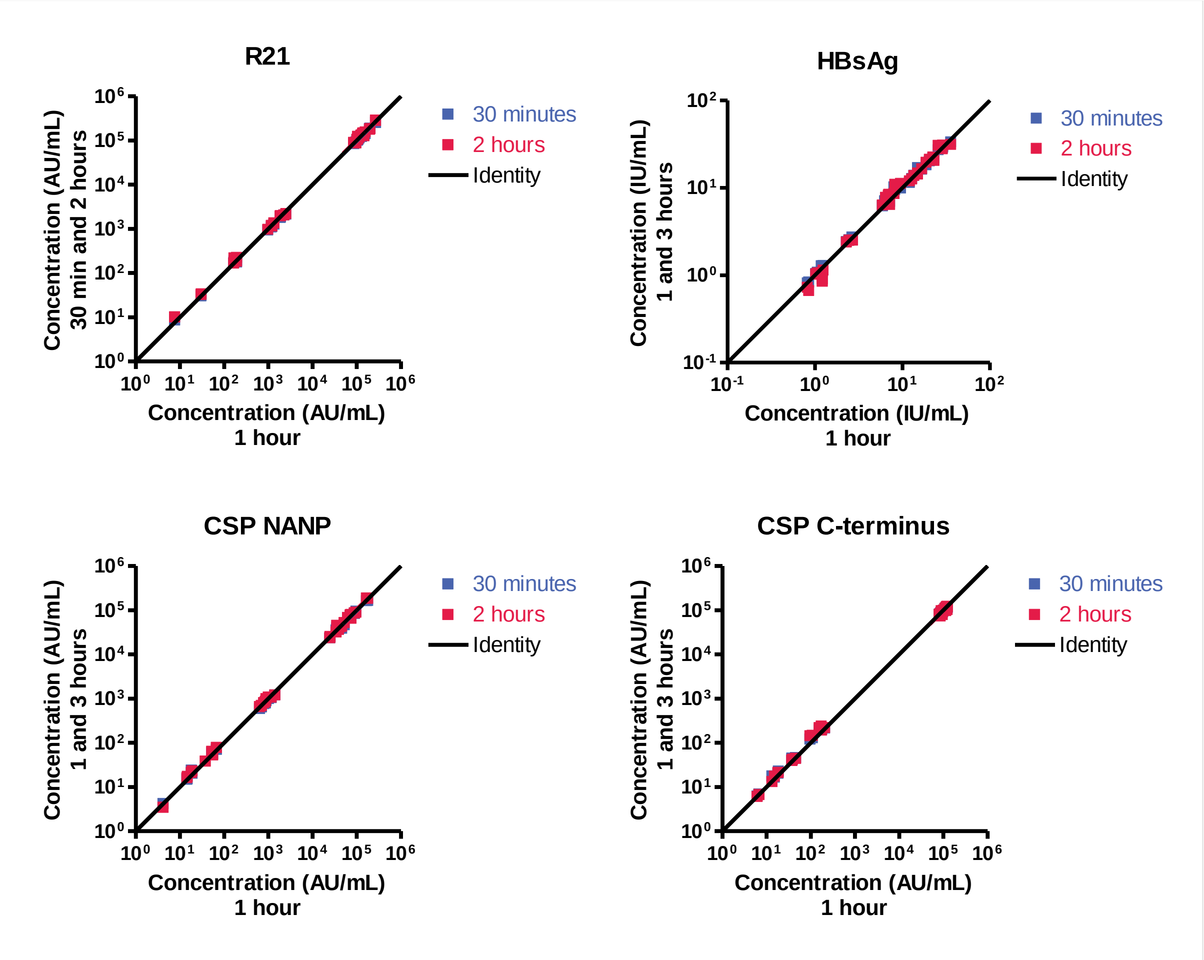

- Sample concentrations from 30 minutes and 2-hour detection incubation times were compared to the standard condition (1 hour)
- Good correlation of sample concentrations was observed for each condition

Supplementary Figure 12 - Detection Titration

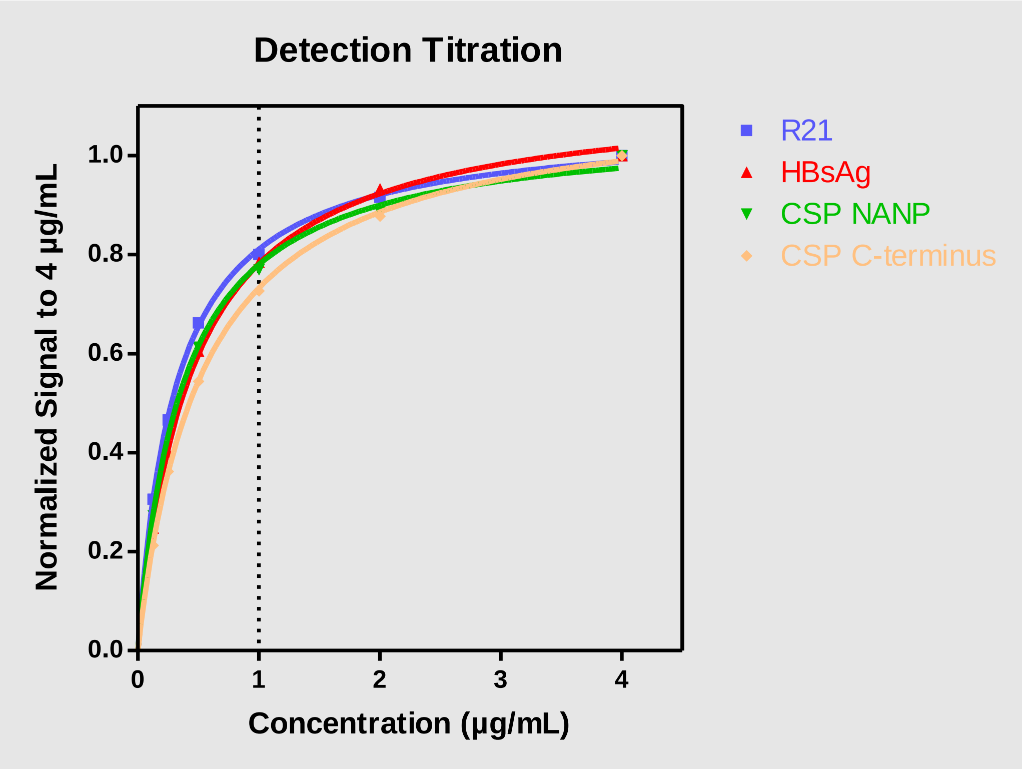

- Signals from each detection concentration were normalized to signals from the 4 µg/mL concentration
- Signals at 1 µg/mL detection (dotted line) reach 70-80% of the signals at 4 µg/mL
- The standard detection concentration of 1 µg/mL is recommended

Conclusions:

- Good correlation of sample concentrations for each condition
- Standard protocol incubation times and conditions are recommended:
    - 30 minutes blocking with Blocker A
    - 2 hour sample incubation
    - 1 hour detection incubation
    - 1 µg/mL detection antibody

Evaluate signal, concentration and recovery at 5 dilutions ranging from 100-fold to 1,000,000-fold in 10-fold steps for all 120 serum and plasma samples and to select the recommended sample dilution factors

- The standard serology assay protocol was followed using the production plate lot

- Blocking with Blocker A Solution for 30 minutes
- Sample incubation for 2 hours
- Detection incubation for 1 hour using
  - SULFO-TAG™ labeled anti-human IgG detection was used at 1 µg/mL
- 24 samples were run each day for 5 days
  - 8 samples per plate
  - 3 plates per day
  - 120 samples were run on 5 days of testing on 15 total plates
  - For each plate, an 8 point standard curve was prepared from the human reference standard serum sample diluted in Diluent 100 to 10,000-fold top of curve (TOC) and serial diluted in 4-fold steps

Results:

- Concentrations were measured for all 120 samples; at least two sample dilutions are recommended to cover the full range of titers
  - 1,000-fold dilution is recommended for D0 and D35 samples
  - 100,000-fold dilution is recommended for D84 and D196 samples

Supplementary Figure 13 - Dilution Linearity Standard Curves

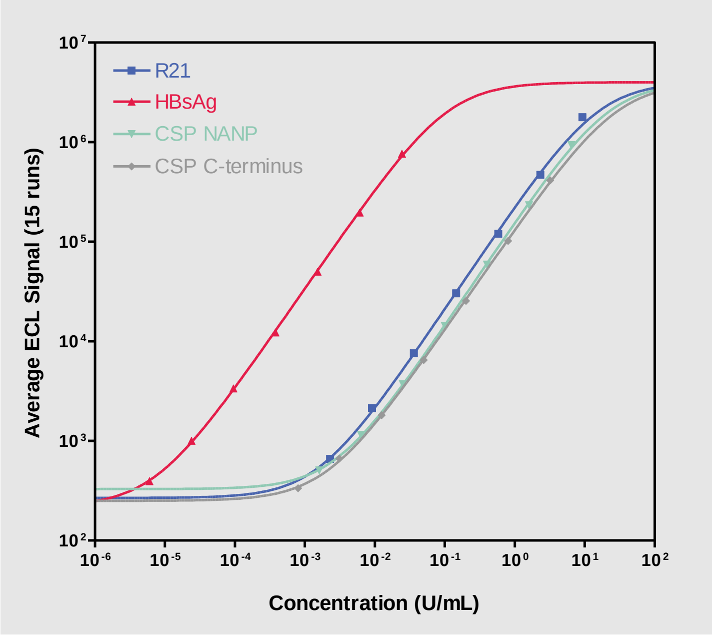

- Average ECL signals from 15 plates run in duplicate were plotted and shown below
- Good standard curve performance for all four assays

Supplementary Table 16 - Dilution Linearity Standard Curves

	R21		HBsAg		CSP NANP		CSP C-terminus	
	AU/mL	Signal	IU/mL	Signal	AU/mL	Signal	AU/mL	Signal
Std 1	9.3000	1782431	0.024300	765211	6.4000	939385	3.2000	414668
Std 2	2.3250	470502	0.006075	197257	1.6000	232648	0.8000	102266
Std 3	0.5813	120367	0.001519	50312	0.4000	59246	0.2000	25566
Std 4	0.1453	30412	0.000380	12293	0.1000	14326	0.0500	6520
Std 5	0.0363	7616	0.000095	3375	0.0250	3710	0.0125	1817
Std 6	0.0091	2137	0.000024	1009	0.0063	1150	0.0031	668
Std 7	0.0023	661	0.000006	395	0.0016	507	0.0008	336
Std 8	0.0000	138	0.000000	122	0.0000	215	0.0000	146
Hill Slope	0.99		0.98		1.00		0.98	
LLOD	0.0021		0.000011		0.0036		0.0032	

Supplementary Table 17 - Dilution Linearity: Standard Curve Statistics

	Intra-plate CV (%)				Inter-plate CV (%)			
	R21	HBsAg	CSP NANP	CSP C-terminus	R21	HBsAg	CSP NANP	CSP C-terminus
Std 1	1.8	3.7	2.4	2.3	3.8	3.9	5.1	2.4
Std 2	1.6	2.0	1.9	1.0	4.8	2.7	4.9	1.7
Std 3	2.6	5.1	1.8	1.7	3.8	2.6	4.8	3.6
Std 4	2.6	4.1	3.6	2.5	8.1	7.2	9.6	7.8
Std 5	3.4	7.5	4.4	8.4	13.1	12.6	14.5	14.9
Std 6	10.4	20.7	19.2	37.6	18.2	19.5	21.5	24.0
Std 7	29.1	56.0	46.5	62.6	23.1	30.7	35.1	42.0
Std 8	162.9	257.8	90.9	146.4	64.5	75.6	57.3	68.1
								<20%

	Recovery (%)							
	R21		HBsAg		CSP NANP		CSP C-terminus	
	Average	Range	Average	Range	Average	Range	Average	Range
Std 1	101	99 - 106	104	100 - 113	103	99 - 114	108	100 - 125
Std 2	101	96 - 105	102	98 - 106	100	94 - 103	101	96 - 107
Std 3	101	97 - 108	100	96 - 103	101	96 - 107	97	93 - 102
Std 4	100	95 - 104	93	87 - 98	97	86 - 104	94	83 - 103
Std 5	97	89 - 101	96	88 - 106	96	85 - 105	94	79 - 106
Std 6	101	94 - 107	101	95 - 108	101	96 - 109	113	97 - 138
Std 7	104	94 - 120	120	81 - 165	140	81 - 322	239	89 - 567
Std 8	NA	NA	NA	NA	NA	NA	NA	NA
								70-130%

- Intra-plate and inter-plate CVs <20% are highlighted
    - CVs for Std 1 to Std 5 are <15%
- Average recovery between 70% and 130% are highlighted

Supplementary Table 18 - Dilution Linearity: Percent of Samples Within Assay Range

D0 Serum Dilution	In Range (%)			
	R21	HBsAg	NANP	CSP C-
100	71	82	59	65
1000	100	100	88	100
10000	88	100	71	71
100000	65	47	59	41
1000000	18	12	6	6

D0 Plasma Dilution	In Range (%)			
	R21	HBsAg	NANP	CSP C-
100	90	80	90	95
1000	100	95	100	100
10000	60	80	45	40
100000	10	30	10	5
1000000	0	10	0	0

D35 Serum Dilution	In Range (%)			
	R21	HBsAg	NANP	CSP C-
100	100	60	100	100
1000	100	80	80	100
10000	60	100	0	20
100000	0	60	0	0
1000000	0	40	0	0

D35 Plasma Dilution	In Range (%)			
	R21	HBsAg	NANP	CSP C-
100	60	60	60	100
1000	100	80	100	100
10000	80	100	80	40
100000	40	80	40	0
1000000	0	40	0	0

D84 Serum Dilution	In Range (%)			
	R21	HBsAg	NANP	CSP C-
100	9	48	9	4
1000	17	78	22	26
10000	87	100	91	83
100000	96	83	96	96
1000000	91	52	87	78

D84 Plasma Dilution	In Range (%)			
	R21	HBsAg	NANP	CSP C-
100	4	8	4	8
1000	8	38	12	8
10000	50	85	54	38
100000	92	92	92	77
1000000	92	81	92	92

D196 Serum Dilution	In Range (%)			
	R21	HBsAg	NANP	CSP C-
100	0	0	0	0
1000	0	75	0	0
10000	17	100	58	0
100000	100	100	100	100
1000000	100	58	100	100

D196 Plasma Dilution	In Range (%)			
	R21	HBsAg	NANP	CSP C-
100	0	0	0	0
1000	0	75	0	0
10000	25	100	83	0
100000	100	100	100	100
1000000	100	75	100	100

≥80%

- 1,000-fold dilution is recommended for D0 and D35 samples
- 100,000-fold dilution is recommended for D84 and D196 samples

Supplementary Figure 14 - Dilution Linearity: R21 and HBsAg Signals

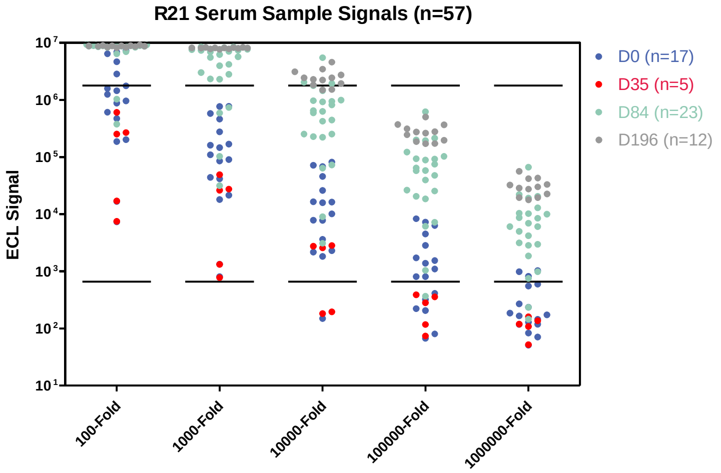

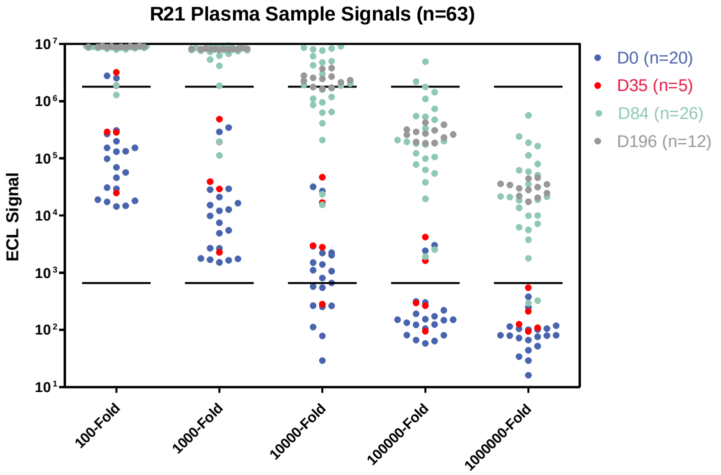

- Std 1 and Std 7 signals (black lines) are indicated
- 1,000-fold dilution is recommended for D0 and D35 samples
- 100,000-fold dilution is recommended for D84 and D196 samples

Supplementary Figure 15 - Dilution Linearity: CSP NANP and CSP C-terminus Signals

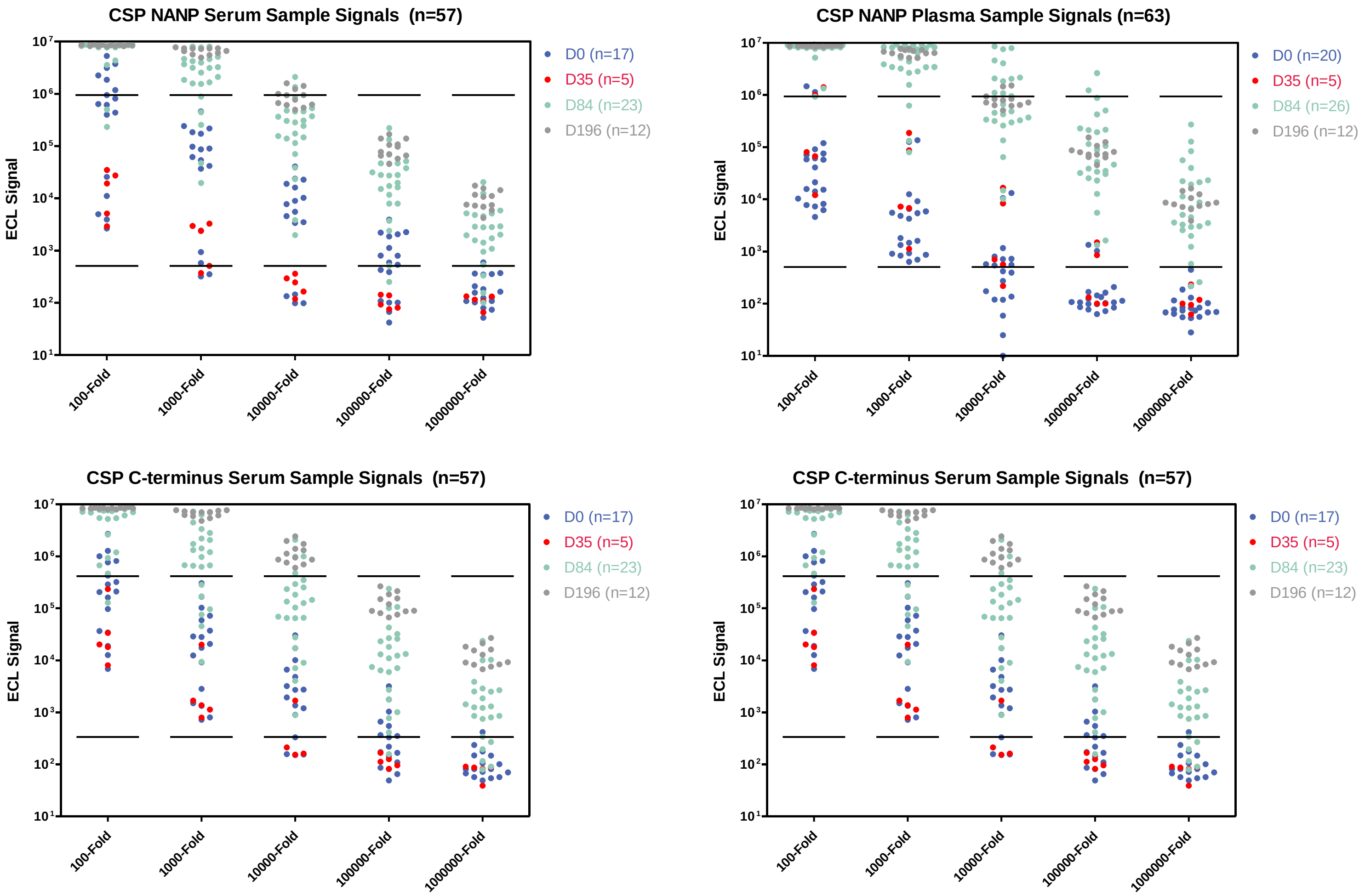

- Std 1 and Std 7 signals (black lines) are indicated
- 1,000-fold dilution is recommended for D0 and D35 samples
- 100,000-fold dilution is recommended for D84 and D196 samples

Supplementary Figure 16 - Dilution Linearity: R21 and HBsAg Concentrations

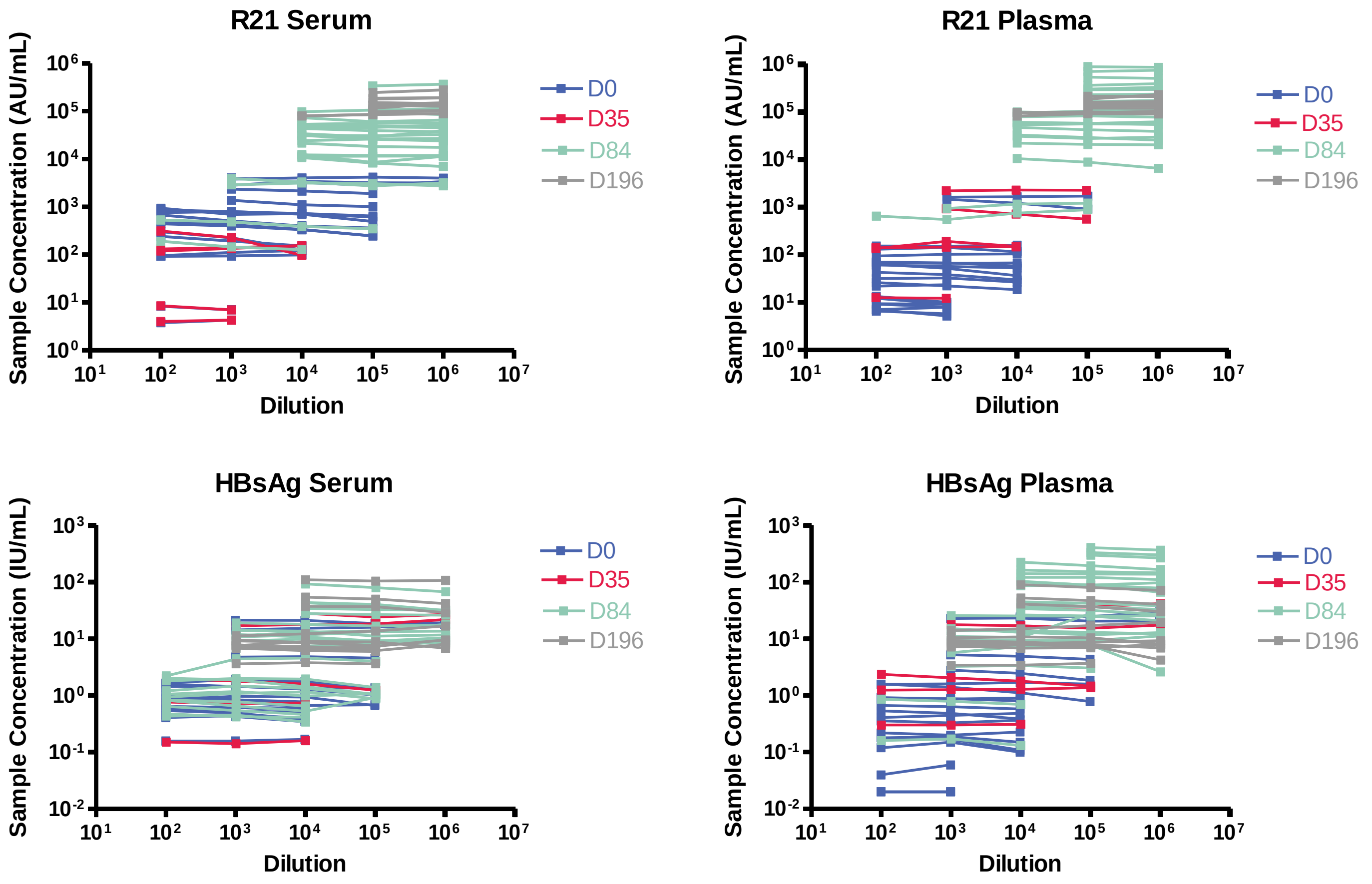

- Sample concentrations within the assay range (between Std 1 and Std 7) were plotted at each dilution
- Concentrations of most samples are linear across 2 or more dilutions

Supplementary Figure 17 - Dilution Linearity: CSP NANP and CSP C-terminus Concentrations

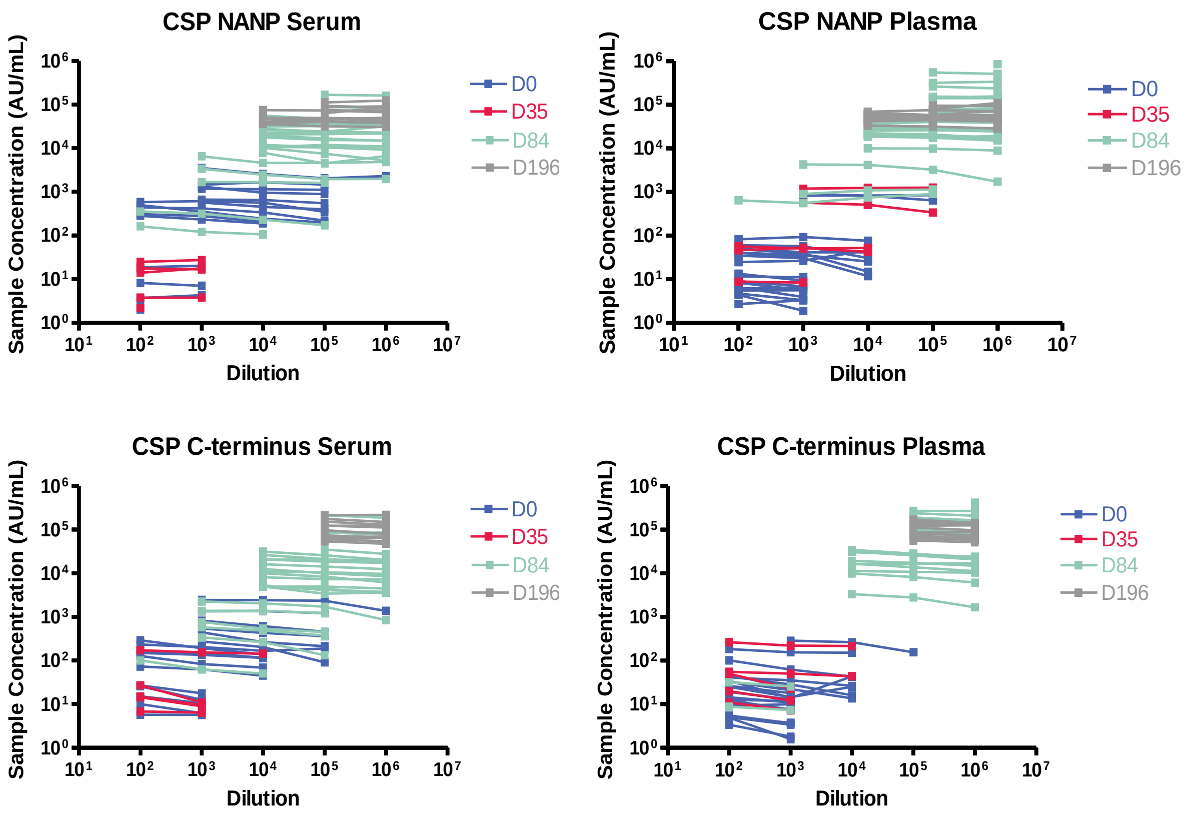

- Sample concentrations within the assay range (between Std 1 and Std 7) were plotted at each dilution
- Concentrations of most samples are linear across 3 dilutions

Supplementary Figure 18 - Dilution Linearity: Sample Concentrations

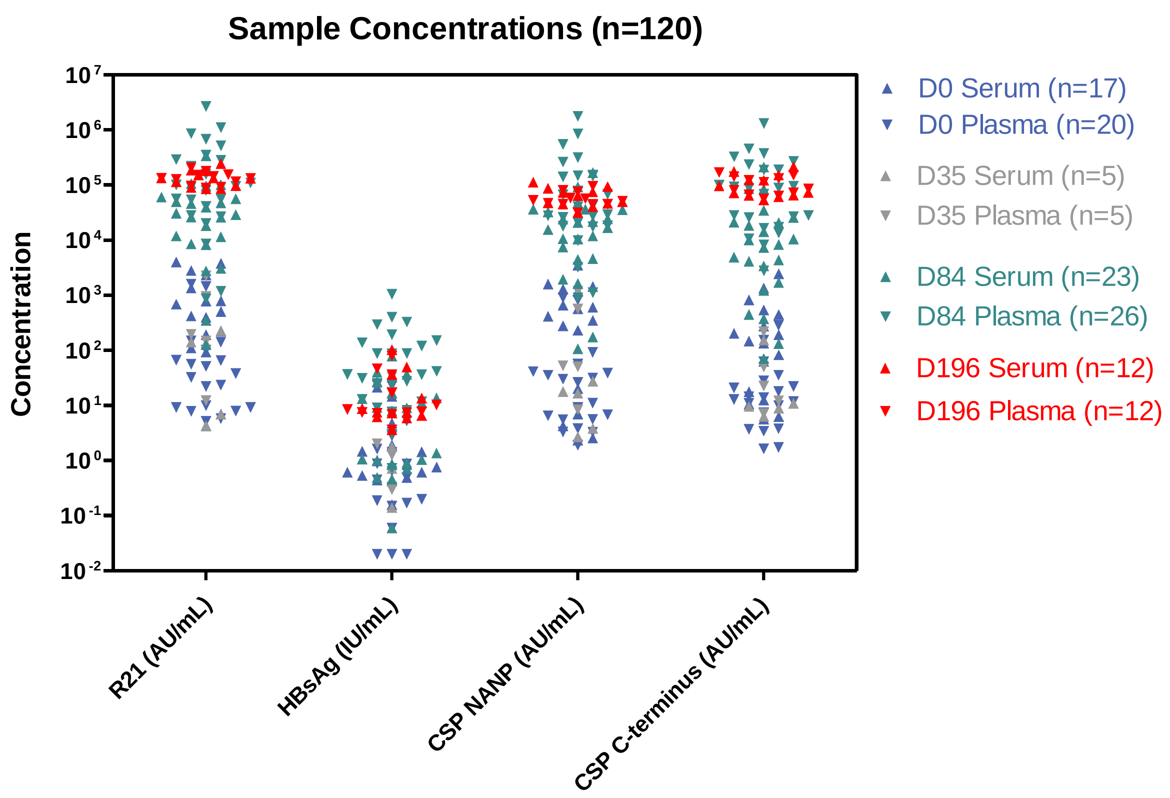

- Concentrations from 1,000-fold dilution of D0 and D35 samples are plotted
- Concentrations from 100,000-fold dilution of D84 and D196 samples are plotted

Reproducibility: Evaluate accuracy (% recovery) and precision (%CVs) of each assay

- The standard serology assay protocol was followed using the production plate lot
  - Blocking with Blocker A Solution for 30 minutes
  - Sample incubation for 2 hours
  - Detection incubation for 1 hour using
    - SULFO-TAG™ labeled anti-human IgG detection was used at 1 µg/mL
- 6 runs consisting of independent preparations of standard curve, controls and samples
  - 2 runs per day for 3 days
  - Human serum reference standard sample was run in duplicate for standard curves

- 3 controls (high, mid and low) prepared from reference standard was pre-diluted and tested neat and run with quadruplicate replicates
- 16 samples run with quadruplicate replicates
  - 2 samples from each of the 4 study time points from each sample matrix (serum and plasma)
  - 8 samples diluted 1000-fold (D0 and D35) and 8 samples diluted 100,000-fold (D84 and D196)

Results:

- Good reproducibility was observed for all assays
- Control and sample recoveries were within 70-130%
- Intra-run and inter-run CVs were < 20%

Supplementary Figure 19 - Reproducibility: R21

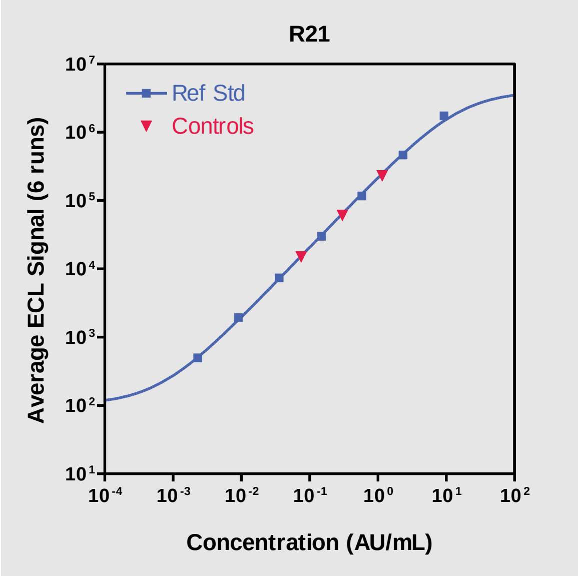

Supplementary Figure 20 - Reproducibility: R21 Controls

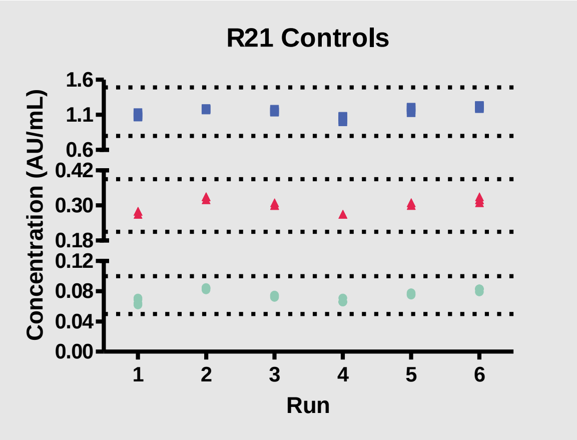

- Intra-run and inter-run CVs were < 20%

Supplementary Table 19 - Reproducibility: R21

R21

Sample	AU/mL	Avg Signal (n=12)	Avg Signal CV (%)	Calc. Conc. (AU/mL)	Avg Intra-run CV (%)	Inter-run CV (%)
Std 1	9.30	1749589	1.9	9.32	2.1	2.6
Std 2	2.33	467380	0.9	2.32	0.9	1.5
Std 3	0.58	116592	2.1	0.57	2.0	2.4
Std 4	0.15	30093	1.1	0.15	1.1	2.3
Std 5	0.036	7402	3.9	0.036	4.0	4.3
Std 6	0.0091	1944	3.1	0.0094	3.2	3.3
Std 7	0.0023	500	3.9	0.0022	4.6	5.7
Std 8	0.00	65	14.7	0.0000	NA	NA
					>20%	

	Min	Max	Median
Hill Slope	0.99	1.03	1.00
LLOD (AU/mL)	0.00031	0.00050	0.00037

Matrix	TP	DF	Sample	Avg Signal (n=24)	Avg Signal CV (%)	Calc. Conc. (AU/mL)	Avg Intra-run CV (%)	Inter-run CV (%)
		1	High Control	232532	2.0	1.15	2.1	5.6
		1	Mid Control	60970	1.9	0.30	1.9	7.3
		1	Low Control	15103	2.9	0.075	2.9	9.4
Serum	D0	1,000	PID 16	120188	3.9	590	3.9	5.8
		1,000	PID 22	633302	4.9	3164	5.1	7.0
	D35	1,000	PID 43	515623	4.4	2564	4.5	6.7
		1,000	PID 83	288801	3.1	1423	3.1	4.4
	D84	100,000	PID 37	91121	4.8	44667	4.8	5.5

	D196	100,000	PID 63	108680	2.8	53310	2.8	4.8
		100,000	PID 19	518703	1.8	257973	1.9	5.1
		100,000	PID 611	261583	3.6	128641	3.6	5.0
Plasma	D0	1,000	PID 77	1500	7.5	7.29	7.7	12.4
		1,000	PID 83	55110	4.8	269	4.8	13.7
	D35	1,000	PID 77	31847	3.9	157	3.9	8.5
		1,000	PID 83	205692	3.2	1010	3.2	6.6
	D84	100,000	PID 37	211626	3.2	104017	3.2	4.2
		100,000	PID 44	221620	5.8	109271	5.8	10.4
	D196	100,000	PID 209	282627	2.7	139348	2.7	5.9
		100,000	PID 611	244868	3.5	120497	3.5	5.7

Supplementary Figure 21 - Reproducibility: HBsAg

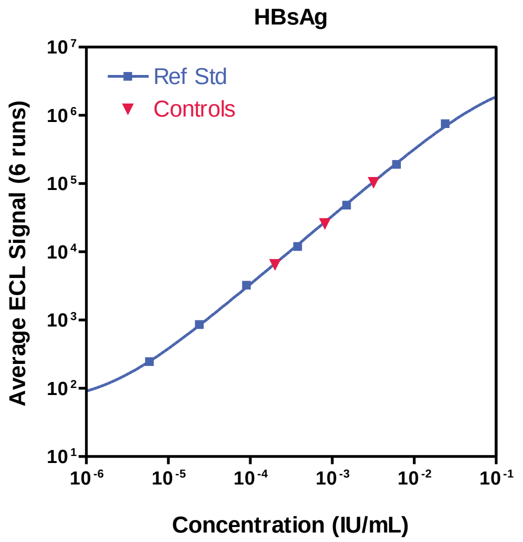

Supplementary Figure 22 - Reproducibility: HBsAg Controls

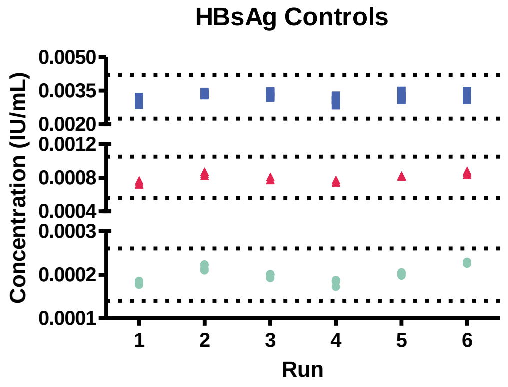

- Intra-run and inter-run CVs were < 20%

Supplementary Table 20 - Reproducibility: HBsAg

HBsAg

Sample	IU/mL	Avg Signal (n=12)	Avg Signal CV (%)	Calc. Conc. (IU/mL)	Avg Intra-run CV (%)	Inter-run CV (%)
Std 1	0.024	756539	2.4	0.024	2.5	2.3
Std 2	0.0061	190581	2.1	0.0060	2.1	2.5
Std 3	0.0015	48572	3.6	0.0015	3.6	3.6
Std 4	0.00038	11985	2.0	0.00037	2.0	3.4
Std 5	0.00009	3248	2.1	0.00010	2.2	3.0
Std 6	0.000024	859	4.0	0.000025	4.2	5.4
Std 7	0.0000059	246	10.6	0.0000056	14.9	13.0
Std 8	0.0000	71	13.8	0.0000	NA	NA
					>20%	

	Min	Max	Median
Hill Slope	0.99	1.03	1.00
LLOD (IU/mL)	0.0000020	0.0000031	0.0000024

Matrix	TP	DF	Sample	Avg Signal (n=24)	Avg Signal CV (%)	Calc. Conc. (IU/mL)	Avg Intra-run CV (%)	Inter-run CV (%)
		1	High Control	103403	4.6	0.0032	4.6	5.7
		1	Mid Control	25804	2.2	0.00081	2.2	6.0
		1	Low Control	6459	2.3	0.00020	2.3	9.4
Serum	D0	1,000	PID 16	15907	2.9	0.50	2.9	5.0
		1,000	PID 22	14643	8.0	0.46	8.1	10.9
	D35	1,000	PID 43	1151421	1.9	38.4	2.0	9.7
		1,000	PID 83	84635	5.0	2.64	5.0	4.9
	D84	100,000	PID 37	10609	4.1	33.0	4.1	4.8
		100,000	PID 63	34883	3.4	109	3.4	3.9
	D196	100,000	PID 19	33465	4.3	104	4.3	5.4
		100,000	PID 611	16089	2.9	50.1	2.9	3.1
Plasma	D0	1,000	PID 77	21065	3.0	0.66	3.0	4.4
		1,000	PID 83	57733	4.3	1.80	4.3	11.1
	D35	1,000	PID 77	29609	1.8	0.92	1.8	5.0
		1,000	PID 83	66893	5.6	2.09	5.6	8.2
	D84	100,000	PID 37	27784	3.8	86.7	3.8	4.6
		100,000	PID 44	4443	3.1	13.8	3.1	9.4
	D196	100,000	PID 209	27137	4.0	84.8	4.0	6.1
		100,000	PID 611	14676	2.4	45.7	2.4	4.0

Supplementary Figure 23 - Reproducibility: CSP NANP

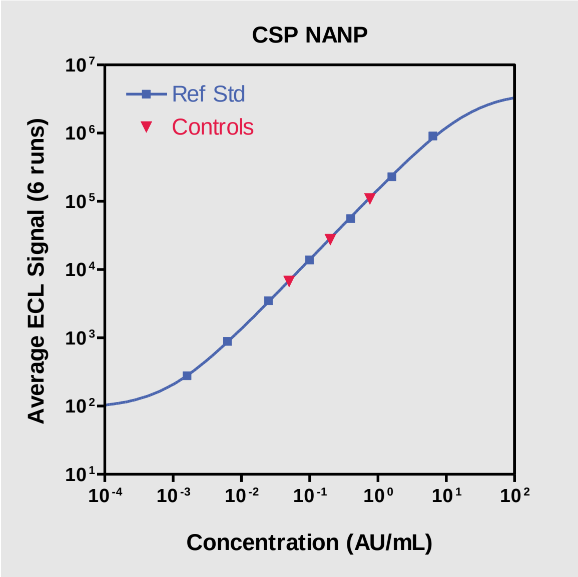

Supplementary Figure 24 - Reproducibility: CSP NANP Controls

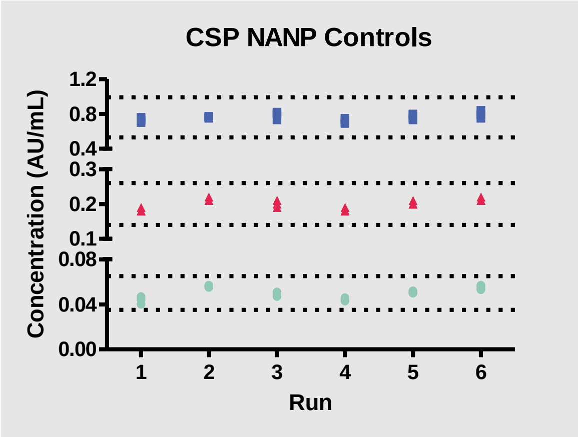

- Intra-run and inter-run CVs were < 20%

Supplementary Table 21 - Reproducibility: NANP

NANP

Sample	AU/mL	Avg Signal (n=12)	Avg Signal CV (%)	Calc. Conc. (AU/mL)	Avg Intra-run CV (%)	Inter-run CV (%)
Std 1	6.40	909834	3.0	6.42	3.2	3.1
Std 2	1.60	229744	1.8	1.59	1.8	2.0
Std 3	0.40	56114	0.9	0.40	0.9	1.8
Std 4	0.10	13807	3.2	0.10	3.1	3.6
Std 5	0.025	3508	5.0	0.026	5.0	5.1
Std 6	0.0063	887	3.6	0.0062	3.8	5.2
Std 7	0.0016	278	8.9	0.0015	14.1	15.2
Std 8	0.00	88	18.0	0.000	NA	NA
					>20%	

	Min	Max	Median
Hill Slope	1.00	1.07	1.02
LLOD (AU/mL)	0.00049	0.00085	0.00058

Matrix	TP	DF	Sample	Avg Signal (n=24)	Avg Signal CV (%)	Calc. Conc. (AU/mL)	Avg Intra-run CV (%)	Inter-run CV (%)
		1	High Control	108855	3.9	0.76	3.8	5.2
		1	Mid Control	27739	2.6	0.20	2.5	6.9
		1	Low Control	6744	3.2	0.050	3.1	9.4
Serum	D0	1,000	PID 16	59132	4.6	418	4.6	6.0
		1,000	PID 22	244554	11.3	1690	11.2	14.3
	D35	1,000	PID 43	196885	3.2	1368	3.2	5.9
		1,000	PID 83	123192	2.2	860	2.1	3.8
	D84	100,000	PID 37	42648	4.9	30330	4.8	6.0
		100,000	PID 63	29149	2.6	20921	2.5	6.2
	D196	100,000	PID 19	161288	2.9	112278	2.9	5.9
		100,000	PID 611	102425	3.5	71707	3.5	4.7
Plasma	D0	1,000	PID 77	533	20.9	3.54	24.6	33.0
		1,000	PID 83	1077	5.3	7.76	5.6	19.7
	D35	1,000	PID 77	5402	2.8	39.9	2.8	11.7
		1,000	PID 83	82364	2.2	579	2.1	5.3
	D84	100,000	PID 37	109654	3.3	76726	3.2	4.6
		100,000	PID 44	53260	6.8	37837	6.7	12.3
	D196	100,000	PID 209	82540	6.3	58081	6.3	8.8
		100,000	PID 611	96475	3.5	67657	3.5	6.3

Supplementary Figure 25 - Reproducibility: CSP C-terminus

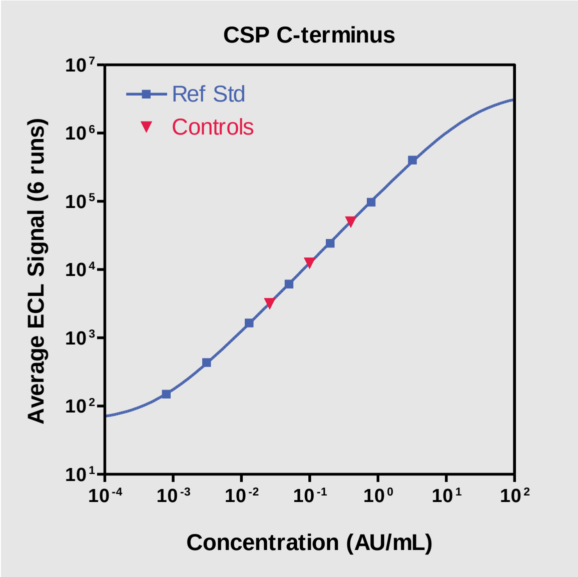

Supplementary Figure 26 - Reproducibility: CSP C-terminus Controls

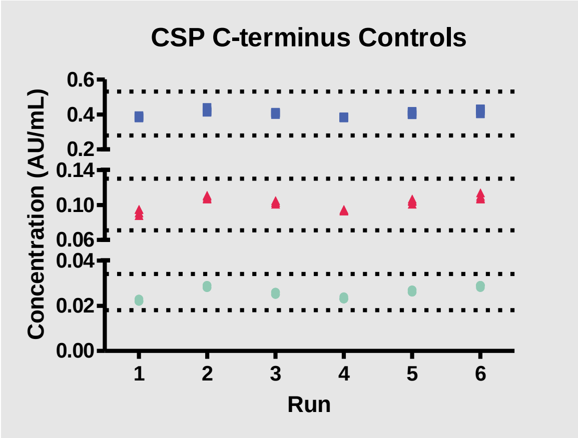

- Intra-run and inter-run CVs were < 20%

Supplementary Table 22 - Reproducibility: CSP C-terminus

CSP C-term

Sample	AU/mL	Avg Signal (n=12)	Avg Signal CV (%)	Calc. Conc. (AU/mL)	Avg Intra-run CV (%)	Inter-run CV (%)
Std 1	3.20	402871	2.1	3.25	2.1	2.0
Std 2	0.80	97400	0.8	0.79	0.8	1.3
Std 3	0.20	24240	0.9	0.20	0.9	2.0
Std 4	0.05	6122	1.4	0.05	1.4	1.8
Std 5	0.013	1647	4.4	0.013	4.6	4.4
Std 6	0.0031	434	7.5	0.0031	8.7	9.8
Std 7	0.0008	150	10.1	0.00076	16.7	17.1
Std 8	0.00	61	34.4	0.00010	NA	NA
					>20%	

	Min	Max	Median
Hill Slope	0.97	1.04	1.01
LLOD (AU/mL)	0.00050	0.00149	0.00065

Matrix	TP	DF	Sample	Avg Signal (n=24)	Avg Signal CV (%)	Calc. Conc. (AU/mL)	Avg Intra-run CV (%)	Inter-run CV (%)
		1	High Control	49919	2.0	0.40	2.0	4.3
		1	Mid Control	12483	2.2	0.10	2.2	7.1
		1	Low Control	3173	2.0	0.026	2.0	9.4
Serum	D0	1,000	PID 16	28912	2.1	235	2.1	2.6
		1,000	PID 22	170534	1.4	1374	1.4	3.8
	D35	1,000	PID 43	6259	2.1	51.1	2.1	6.1
		1,000	PID 83	2260	3.8	18.4	3.9	12.5
	D84	100,000	PID 37	13527	5.5	11014	5.4	5.4
		100,000	PID 63	43656	2.0	35369	2.0	3.1
	D196	100,000	PID 19	255649	2.1	205953	2.1	5.5
		100,000	PID 611	86841	3.9	70113	3.9	5.2
Plasma	D0	1,000	PID 77	18717	2.3	152	2.3	6.1
		1,000	PID 83	1326	2.6	10.5	2.7	11.7
	D35	1,000	PID 77	21749	2.5	177	2.4	8.1
		1,000	PID 83	1809	3.1	14.6	3.2	8.5
	D84	100,000	PID 37	20641	2.4	16796	2.4	3.9
		100,000	PID 44	111047	5.1	89665	5.1	9.5
	D196	100,000	PID 209	138072	3.5	111322	3.4	4.8
		100,000	PID 611	81056	2.7	65486	2.7	4.8

- Conclusions:
    - Good reproducibility was observed for all assays
    - Control and sample recoveries were within 70-130%
    - Intra- and inter-run CVs were < 20%

Supplementary Note 3

Final Assay Protocol

The standard serology protocol was as follows:

- Add 150 µL per well of Blocker A solution
- Seal the plate and incubate for 30 minutes at room temperature with shaking at 700 rpm
- Wash, add 50 µL per well of reference standard, controls and samples diluted in Diluent 100
- Seal the plate and incubate for 2 hours at room temperature with shaking at 700 rpm
- Wash, add 50 µL per well of detection at 1 ug/mL diluted in Diluent 100
- Seal the plate and incubate for 1 hour at room temperature with shaking at 700 rpm
- Wash, add 150 µL per well of MSD Gold Read Buffer B and read the plate immediately using a MESO QuickPlex SQ 120

Reference standard, samples and detection antibody are diluted in Diluent 100

Reference standard is diluted 10,000-fold to the top of curve and serial diluted 4-fold to generate an 8 point standard curve

D0 and D35 samples are diluted 1,000-fold

D84 and D196 samples are diluted 100,000-fold

An intermediate sample dilution of 10,000-fold could also be considered, if desired

Example dilution scheme for 1,000-fold dilutions:

- 10 uL + 390 uL Diluent 100
- 10 uL + 240 uL Diluent 100

Example dilution scheme for 100,000-fold dilutions:

- 10 uL + 990 uL Diluent 100
- 10 uL + 490 uL Diluent 100
- 10 uL + 190 uL Diluent 100

Peptide Sequences

Full length R21 –

MDPNANPNANPNANPNANPNANPNANPNANPNANPNANPNANPNANPNANPNANPNANPNAN

PNANPNANPNANPNKNNQGNGQGHNMPNDPNRNVDENANANSAVKNNNNEEPSDKHIKEYLN

KIQNSLSTEWSPCSVTCGNGIQVRIKPGSANKPKDELDYANDIEKKICKMEKCSSVPVTNMENITS

GFLGPLLVLQAGFFLLTRILTIPQSLDSWWTSLNFLGGSPVCLGQNSQSPTSNHSPTSCPPICPGYR

WMCLRRFIIFLFILLLCLIFLLVLLDYQGMLPVCPLIPGSTTTNTGPCKTCTTPAQGNSMFPSCCCT

KPTDGNCTCIPIPSSWAFAKYLWEWASVRFSWLSLLVPFVQWFVGLSPTVWLSAIWMMWYWG

PSLYSIVSPFIPLLPIFFCLWVYI

NANP

NANPNANPNANPNANPNANPNANPC

C-term

CEPSDKHIKEYLNKIQNSLSTEWSPCSVTCGNGIQVRIKPGSANKPKDELDYANDIEKKICKMEKC

S

ICH Q14 Guideline Requirements for Analytical Procedure Validation

Supplementary Table 23 - ICH Guideline requirements

Validation Parameter	Description	Key Points
Specificity	Ability to assess the analyte unequivocally in the presence of components that may be expected to be present.	Ensure no interference from other components. Demonstrate the method’s ability to measure the analyte in presence of impurities, degradants, matrix, etc.
Linearity	Ability to obtain test results that are directly proportional to the concentration of analyte in the sample.	Test across the range of expected analyte concentrations. Use at least five concentrations. Calculate correlation coefficient, slope, and intercept.
Range	The interval between the upper and lower levels of analyte that have been demonstrated to be determined with precision, accuracy, and linearity.	Establish by applying the method to samples with varying concentrations.
Accuracy	The closeness of agreement between the value found and the value that is accepted as a reference standard.	Use spiked samples or known concentrations. Evaluate recovery percentage.

Precision	The degree of agreement among individual test results when the procedure is applied repeatedly.	Include repeatability, intermediate precision, and reproducibility. Evaluate standard deviation or relative standard deviation.
Detection Limit (LOD)	The lowest amount of analyte that can be detected but not necessarily quantified.	Establish using signal-to-noise ratio, typically 3:1. Alternatively, use visual evaluation or standard deviation of the response and slope.
Quantitation Limit (LOQ)	The lowest amount of analyte that can be quantitatively determined with suitable precision and accuracy.	Establish using signal-to-noise ratio, typically 10:1. Alternatively, use visual evaluation or standard deviation of the response and slope.
Robustness	The ability of the method to remain unaffected by small, deliberate variations in method parameters.	Evaluate the impact of variations in method parameters (e.g., pH, temperature, flow rate).
System Suitability	Tests to ensure the system is operating correctly before or during the analysis.	Perform tests such as resolution, repeatability, and theoretical plates.
Stability	Stability of analyte in the sample under analysis conditions.	Test for stability under various conditions (e.g., room temperature, refrigerated, frozen).

Comparisons between NANP peptide repeats

Supplementary Figure 27 - Time course of NANP IgG response using NANP6, NANP12, and NANP19 from n=40 vaccinated Burkinabe infants (VAC076). V1, V2 and V3 are the primary R21 vaccination schedule, with the 1st Booster vaccine shown one year post primary series.

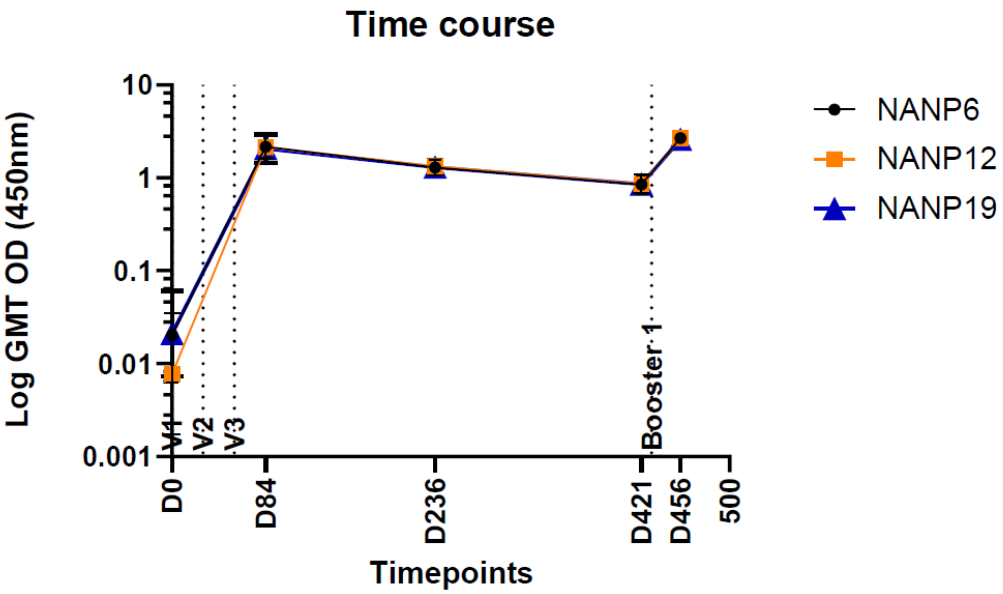
Time course
Log GMT OD (450nm)
10
1
0.1
0.01
0.001
V1
V2
V3
Booster 1
D0
D84
D236
D421
D456
500
Timepoints
NANP6
NANP12
NANP19
